# A Role for Neutrophils in Viral Respiratory Disease

**DOI:** 10.3389/fimmu.2017.00550

**Published:** 2017-05-12

**Authors:** Jeremy V. Camp, Colleen B. Jonsson

**Affiliations:** ^1^Institute of Virology, University of Veterinary Medicine at Vienna, Vienna, Austria; ^2^Department of Microbiology, University of Tennessee-Knoxville, Knoxville, TN, USA

**Keywords:** neutrophil, influenza, acute respiratory distress syndrome, respiratory virus, viral microenvironment

## Abstract

Neutrophils are immune cells that are well known to be present during many types of lung diseases associated with acute respiratory distress syndrome (ARDS) and may contribute to acute lung injury. Neutrophils are poorly studied with respect to viral infection, and specifically to respiratory viral disease. Influenza A virus (IAV) infection is the cause of a respiratory disease that poses a significant global public health concern. Influenza disease presents as a relatively mild and self-limiting although highly pathogenic forms exist. Neutrophils increase in the respiratory tract during infection with mild seasonal IAV, moderate and severe epidemic IAV infection, and emerging highly pathogenic avian influenza (HPAI). During severe influenza pneumonia and HPAI infection, the number of neutrophils in the lower respiratory tract is correlated with disease severity. Thus, comparative analyses of the relationship between IAV infection and neutrophils provide insights into the relative contribution of host and viral factors that contribute to disease severity. Herein, we review the contribution of neutrophils to IAV disease pathogenesis and to other respiratory virus infections.

## Introduction

Neutrophils are a type of polymorphonuclear granulocyte that differentiate from myeloblasts in the bone marrow to comprise approximately 60% of the circulating blood leukocytes (1). The formation of intracellular granules (azurophilic granules, specific granules, gelatinase granules, and secretory vesicles) and the morphologically characteristic segmentation of nuclei occur during the terminal differentiation process into neutrophils (1). Neutrophils are often considered professional bacteria-responsive immune cells: they express bacteria-specific receptors (e.g., formylated peptide receptors or certain toll-like receptors, “TLRs”) and their granules have anti-bacterial or bacteriostatic properties. Currently, their role in viral infection has received very little scientific attention (2).

**Figure 1:**
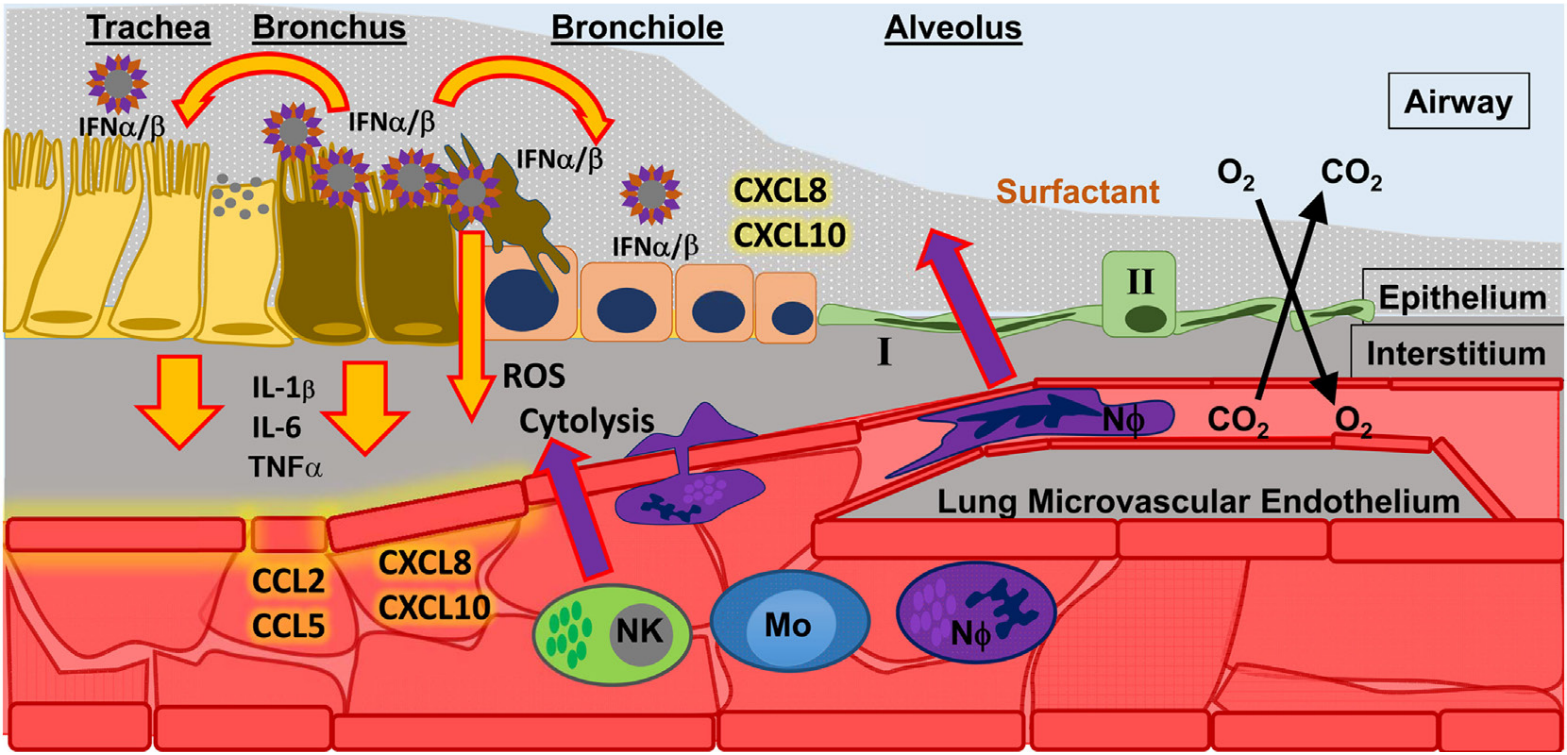
**Influenza A virus (IAV) infection in the upper respiratory tract**. Infection of epithelial cells in the bronchus results in the release of type I interferons (IFN α/β) which signal to nearby cells. The result of IFN α/β signaling is the release of pro-inflammatory cytokines (e.g., IL-1β, IL-6, TNFα) that signal to endothelial cells, which help spread inflammatory signals (chemokines, such as CCL2, CCL5, CXCL8, CXCL10) throughout the blood to recruit innate immune cells to the site of infection. Recruited innate immune cells [such as natural killer cells (NK); monocytes (Mo); and neutrophils (NΦ)] must interact with activated endothelium to leave the blood stream and migrate toward the site of infection. There they can perform effector functions to control infection, such as releasing reactive oxygen species (ROS) and directly killing infected cells (cytolysis).

Neutrophils are present during many types of lung diseases associated with acute respiratory distress syndrome (ARDS) and may contribute to acute lung injury (3–12). The lung has a global inflammatory response to infection regardless of etiology, and this response includes the infiltration of neutrophils and macrophages in response to chemotactic signaling which originates in the lung (3, 5, 12–20). These phagocytic cells leave circulation and hone to sites within the infected airways where they may deploy potent effector functions to control disease (Figure 1) in response to pathogen associated molecular patterns (PAMPs) and inflammatory cytokines and chemokines (21, 22). In the case of viral infections, the type I interferons (IFN) and IFN-stimulated genes (ISGs) signal an appropriate immune response (23–26). Lethal infections may result from insufficient information or incorrect information about the specific cause(s) of infection, thereby signaling inappropriate (incorrect or excessive) immune responses (27). Neutrophils, as first-responders to many forms of airway infection, may be a keystone species in determining viral disease outcome; however, neutrophils are poorly studied with respect to viral infection and specifically to respiratory viral disease.

Much research has focused on the role of neutrophils in severe versus mild respiratory disease, as well as their role in bacterial infections. It is, therefore, important to establish a general role of neutrophils in respiratory virus infection to provide a groundwork into more specific questions (e.g., are neutrophils capable of catering to a virus-specific response?). Herein, the evidence for the presence and activities of neutrophils during respiratory viral infection is reviewed. Having established spatio-temporal aspects of the neutrophil response to influenza A virus (IAV), the potential antiviral function(s) of neutrophils during acute virus infection as well as recovery from infection are discussed. The discussion will focus on IAV and neutrophil activities during the course of a “typical” flu infection: activation, migration, and effector functions *in situ* (Figure 1). IAV are particularly well-suited for the study of neutrophils in viral respiratory disease, since they are well-studied in humans and animal models, and it is well-established that infection with specific viral variants (i.e., genetic point mutations) alter the course of disease from mild to severe (28). More recently, specific IAV viral variants that affect pathogenicity have been linked to alterations of the neutrophil response (29). Thus, a comparison of the neutrophil response between disease phenotypes of a single virus species (*Influenza A virus*) may elucidate a role for neutrophils in the viral microenvironment. Herein, we review evidence of neutrophil responses during the course of disease in various IAV phenotypes in animal models of infection, as well as comparing these responses to what is known about neutrophil responses during bacterial infection of the airways.

## Influenza A Viral Phenotypes

Influenza A virus poses a concern for global public health due to emergence of strains with increased human transmission and/or increased pathology (30–35). In 2009, a novel virus type, influenza A(H1N1)pdm09 IAV, emerged with an increased transmission rate and greater disease, i.e., moderate to severe pathology relative to seasonal human IAV (36–50). Clinical isolates of influenza A(H1N1)pdm09 have relatively little genetic variability yet cause variable clinical outcomes from moderate to severe pathology, including ARDS (39, 41, 47, 48, 51). Therefore, they are well-suited to understand host and viral contributions to IAV pathogenesis. A detailed discussion of the viral replicative cycle is beyond this review, yet excellent reviews are plentiful [e.g., Ref. (52–54)]. IAV is a well-studied model for virus infection in laboratory animals, such as mice and ferrets, and much is known about the contributions of viral and host determinants to severe disease (55–58). Retrospective and experimental infection studies routinely demonstrate common occurrences in the formation of severe IAV [including severe influenza pneumonia (SIP) and ARDS] in humans, ferrets, and mice; these include increased cytokine secretions in the lung, diffuse alveolar damage (“DAD,” bronchointerstitial pneumonia in veterinary pathology), and neutrophilic infiltration (29, 55, 58–66).

In general, IAV infection is an excellent model to investigate the respiratory system’s immune response to viral infection, specifically the pathway leading to severe pneumonia and/or ARDS. Influenza disease is commonly relatively mild and self-limiting, although highly pathogenic forms exist (42, 59, 67–72). The major complication from IAV infection is the formation of SIP which may develop into ARDS (59, 65, 67, 68, 70–73). The reason(s) why infection with IAV may lead to severe viral pneumonia and ARDS is poorly understood, but is thought to involve both host and viral factors. The respective and combined contributions of the host innate immune response and viral factors to the timing and severity of SIP are poorly understood. Neutrophils are present in the respiratory tract during infection with mild seasonal IAV, SIP, and highly pathogenic avian influenza viruses [“HPAI,” which includes avian influenza A (H5N1) virus] (50, 51, 65, 67, 68, 74–76). During SIP and HPAI infection, an increase in the number of neutrophils in the lower respiratory tract (LRT) is correlated with disease severity (50, 51, 65, 67, 68, 76).

Although clinical pathology suggests that a spectrum of disease results from IAV infection, there are at least three disease “phenotypes” caused by infection with IAV, listed by increasing case fatality rate: a mild upper respiratory tract (URT) infection, a SIP which can lead to ARDS, and a LRT infection which can lead to hypercytokinemia. The virological basis for disease phenotype is related to adaptations to mammals—most important are receptor specificity and efficiency of replication—and the major mechanisms have been defined through the use of experimental animal models (30, 32, 33, 57, 77–80). An “ideal” viral infection (i.e., one that is successful for the virus and non-lethal for the host) may be considered a balance between virus replication and an immune response necessary to promote viral shedding, typical of mild seasonal (“epidemic”) IAV. In general, emergent IAV, directly or indirectly from avian enzootic cycles, have increased pathology in humans, the most fatal form of which is a syndrome of complete immune dysregulation (65, 69, 70, 81–84). IAV is genetically highly variable, and mechanisms for increased disease severity are multifactorial, involving host and viral factors.

### Uncomplicated Influenza

**Figure 2:**
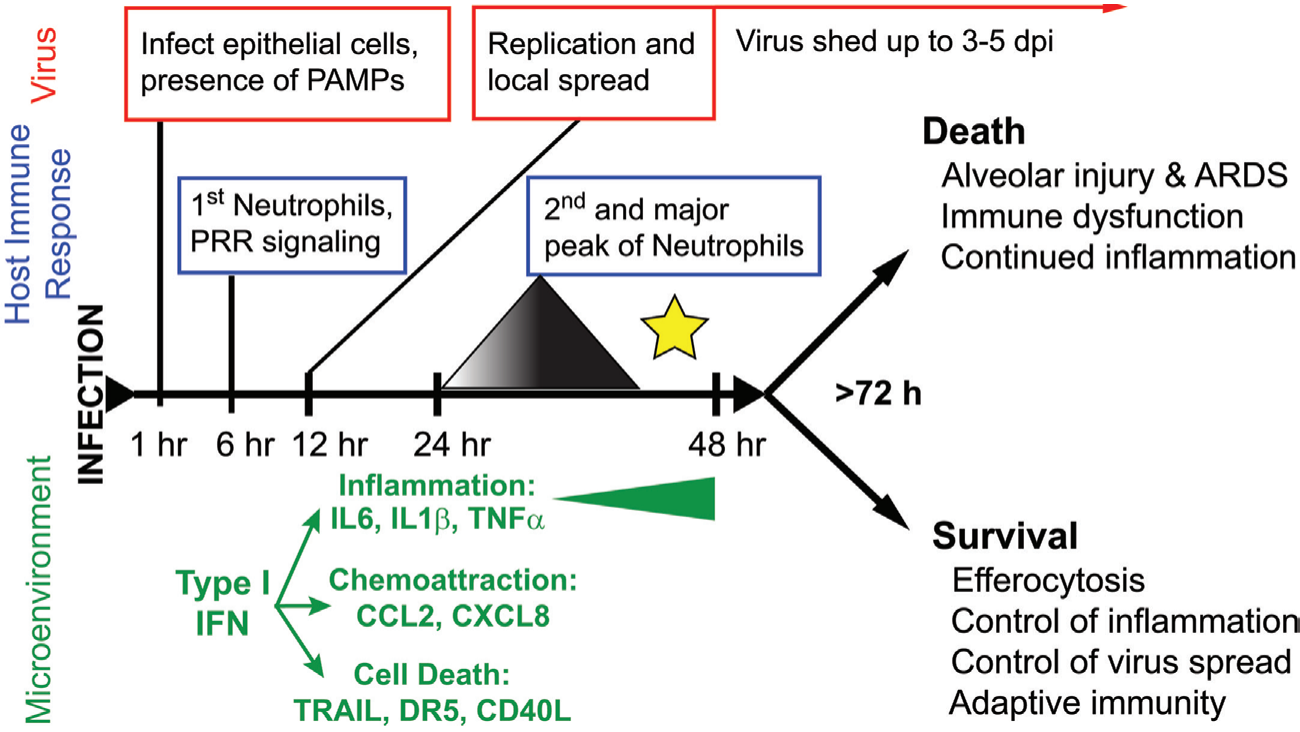
**The course of disease following influenza A virus (IAV) infection**. A timeline depicting major events in the viral replication cycle (red), the host immune response (blue), and the effects on the host tissue environment (green) during an IAV infection of the airways. A star marks the critical point for the formation of severe disease versus recovery from infection. This review posits that at this timepoint, coincident with a second wave of increasing neutrophilia and inflammation, the outcome of disease is determined.

The majority of yearly, seasonal IAV infections in the world cause a relatively mild, self-limiting URT disease. Influenza disease is characterized by an abrupt onset fever, myalgia, and malaise, with symptoms similar to other URT infections, such as sneezing, coryza, and rhinorrhea (67, 85). Symptoms can last anywhere from 1–5 days and are clinically indistinguishable from other “flu”-like illnesses, including bacterial and viral infections that cause the common cold [e.g., *Streptococcus pneumoniae, Haemophilus influenzae*, human rhinovirus (HRV) infection, *Human respiratory syncytial virus* (hRSV) infection, and coronavirus infection] (85–88). Experimental infection of humans with IAV suggests that the virus is mainly restricted to the URT, although sampling the LRT is difficult (67, 68, 87, 89). While fever typically begins 2 days postinfection, virus is shed from the URT in nasal secretions as quickly as 24 h postinfection, allowing efficient transmission prior to symptom onset and continues until 4–5 days postinfection (86, 87, 89) (Figure 2). Rhinorrhea is coincident with neutrophilic rhinitis and shedding of necrotic nasal epithelium (67, 90, 91). Surprisingly, the LRT seems to be involved in uncomplicated IAV infection, although this observation is frequently overlooked or unaddressed in studies (68, 92). In humans, local and systemic concentrations of IL6, CXCL8/IL8, and MCP1/CCL2 correlate with increased disease severity (i.e., symptom severity and increased virus shedding) (87–89, 93) (Figure 2).

### Severe Influenza Pneumonia

Influenza A(H1N1)pdm09 virus spread quickly throughout the globe, much like previous pandemic viruses, such as the 1918 H1N1 “Spanish flu” IAV. Humans infected with influenza A(H1N1)pdm09 virus also presented with typical flu-like symptoms (e.g., fever, cough); however, there was an increased number of cases presenting with dyspnea, respiratory distress, and pneumonia (36–38, 40–48, 50, 94, 95). Additionally, retrospective assessments show a proportionately greater number of adolescents and adults with severe disease compared to typical seasonal influenza, and patients with comorbidities, such as obesity and asthma, were at higher risk of severe infection (51, 96–98). In general, the virus causes infection of URT, as well as bronchitis and bronchiolitis, and a high proportion of cases presented with severe disease in the form of viral pneumonia (42, 51, 96). Histopathologic changes in autopsies revealed extensive cytonecrosis, desquamation, and inflammatory infiltration of the bronchus and trachea, mild to severe necrotizing bronchiolitis (42, 50, 51, 99). The primary pathologic finding of SIP was sporadic to DAD with hyaline membrane formation, edema, and occasionally hemorrhage (42, 50, 51, 99). As is typical of influenza infections, some patients experienced bacterial coinfection although this was not in a majority of patients, including those dying from ARDS (42, 48, 50, 51, 93, 99–103). This may distinguish the influenza A(H1N1)pdm09 virus from the 1918 H1N1 IAV, for which bacterial superinfection was determined to cause a majority of the deaths (104, 105), although this may more accurately reflect improved hygiene and standard of care. As discussed below, studies of the reconstructed 1918 H1N1 IAV using animal models suggest that this virus was highly pathogenic irrespective of secondary bacterial pneumonia (62, 106–108).

Across many cohorts of clinical patients, serum concentration of IL6, CCL2, and CXCL8 were significantly elevated in severe cases of influenza A(H1N1)pdm09 viral pneumonia compared to patients with other confirmed illnesses including seasonal IAV, milder forms of influenza A(H1N1)pdm09 virus, bacterial pneumonia, or other viral respiratory infection [hRSV, HRV, human adenovirus (hAdv)] (93, 100–102, 109). These cytokines and chemokines remained elevated over time (up to 6 days following hospital admission) in cases of severe influenza A(H1N1)pdm09 viral pneumonia, whereas they decreased as patients recovered from seasonal and mild influenza A(H1N1)pdm09 virus infection (93, 100). In severe cases of influenza A(H1N1)pdm09 virus infection, decreased type I IFN and ISG production was occasionally noticed compared to adult patients with seasonal IAV infection (93). The influenza A(H1N1)pdm09 viruses have received much scrutiny, and a large dataset of the genetics and pathogenic phenotypes of virus isolates exists in human and animal models. The H1N1 subtype IAV are highly important viruses due to their pandemic potential, as supported by the historical record (62, 68, 110). Some influenza A(H1N1)pdm09 viruses can infect the LRT in humans and in the ferret animal model, which makes them excellent laboratory viruses to investigate the involvement of the LRT in pathogenesis, specifically in the development of severe disease (42, 111–115). Their overall genetic similarity makes for excellent comparison studies between natural clinical isolates, and reverse genetics systems exist to study molecular pathogenicity (41, 95, 113, 116–126).

### HPAI and Cytokine Storm

Avian IAV has been recorded sporadically entering the human population over the last 20 years, beginning with an H5N1 subtype virus which emerged in China in 1997 (70, 82, 127, 128). It is likely that this avian influenza A (H5N1) virus and other emergent strains result from contact with infected domestic poultry that are infected with HPAI (82). The disease caused by avian influenza A (H5N1) virus is characterized by DAD, alveolar necrosis, and alveolar hemorrhage [human disease, including pathology, is reviewed in Ref. (83)]. There is evidence of viremia and systemic spread; IAV antigen has been detected in the trachea, bronchi, and alveolar pneumocytes (69, 76), as well as infrequently in the brain and gastric epithelium (59, 76, 83). The innate cellular immune response in the lungs was characterized by an increase in inter-alveolar macrophages/histiocytes (59, 65, 69, 71, 81, 129) and only moderate infiltration of lymphocytes and neutrophils in the few patients that were analyzed postmortem (69, 76). Systemically, patient serum had high concentrations of CXCL10, CCL2, IL6, IL8, and IL10 compared to matched-control patients with seasonal H3 and H1 IAV, and these concentrations were correlated with viral load in throat (59, 65, 69). In lethal cases, the result of infection and immune dysregulation led to multiple organ failure (e.g., kidney tubular inflammation, necrotic lesions in brain, impaired liver function) and abnormal clotting. Reactive histiocytes undergoing hemophagocytosis were frequently found in bone marrow and lungs of patients, which is indicative of diseases involving hypercytokinemia (59, 65, 70, 81, 83, 130).

Other events involving avian IAV transmission to humans are known and are often associated with veterinary or other animal workers; for example, a 2004 case of avian influenza A (H7N7) virus infection in a veterinarian in Europe showed severe fatal pneumonia and DAD (131). It was reported that 1 L of serosanguineous fluid was drained from his chest upon autopsy. In 2013, another avian IAV emerged in Southeast Asia; this time an avian influenza A (H7N9) virus (71, 72, 129, 132). The histopathology was similar to avian influenza A (H5N1) virus: severe pneumonia, DAD, and epithelial necrosis were common features of infection with both viruses (71, 72). Therefore, it seems the typical presentation of human patients infected with either virus includes high levels of CXCL10, CCL2, IL-6, and CXCL8 in the plasma, peripheral blood leukopenia, and lung neutrophilia (65, 69, 81, 129, 130, 132), and this is also similar to experimental infection of laboratory animal models (111, 133). There were slightly more bacterial coinfections in cases of H7N9 compared to H5N1 avian IAV (71, 72, 81). In a direct comparison, serum from patients with infected with avian influenza A (H5N1) virus had higher concentrations of IFNα and IFNγ in the blood and lower levels of IL8, whereas the opposite was true for patients with avian influenza A (H7N9) virus (134). Similarly, CXCL9 and CXCL10 were higher in patients with avian influenza A (H5N1) virus, whereas CCL4 concentrations were higher in patients with avian influenza A (H7N9) virus (134). Infection with either virus resulted in higher blood C-reactive protein (CRP) (129). Variability between patients may account for the apparent discrepancies between specific immune responses. Therefore, experimental infection of laboratory animals removes individual variability and provides a clear picture of general disease progression, with important caveats for their comparison to human disease as discussed in more detail in the next section (58, 135).

## Neutrophils in IAV Course of Disease

Neutrophils are increased in the lungs and blood after infection with pathogenic IAV in mice, humans, and ferrets (28, 136, 137). Cell depletion studies have demonstrated that neutrophils are necessary for recovery from severe, but not mild, IAV infection (29, 138, 139). Studies in mice show that neutrophils have effects during both early and late stages of disease (140). As discussed in detail below, initial pathogen sensing through various pathogen recognition receptors (PRRs) stimulates inflammatory signals from resident macrophages to initiate neutrophil chemotaxis to the infected airways (Figure 1). For example, TLR7 recognition of IAV dsRNA- and Myd88-mediated release of TNFα and CCL3 by mononuclear cells is important for neutrophil recruitment to the site of infection (141, 142). Transgenic mice have been used to study the contribution of specific cytokines and chemokines to inflammation following IAV infection, particularly as this relates to “hypercytokinemia,” and are summarized in Tables S1–S4 in Supplementary Material. The signals from the infected lung are propagated systemically by endothelial cells, which recruit and tether neutrophils. The importance of endothelial signaling in the development of severe disease has been shown recently using sphingosine-1 phosphate agonist to prevent severe disease in animal models of both influenza virus and respiratory syncytial virus (143, 144). The complex interactions governing neutrophil extravasation, migration through the interstitium, and crossing the alveolar epithelium are well known in relation to many forms of ALI and the development of ARDS with the exception of conditions surrounding viral infection, although mechanistically they should be quite similar (3–5, 7, 145, 146).

### Neutrophil Migration

In the ferret model, the migration of neutrophils to the lungs occurs in two distinct waves: a first wave within hours of challenge, peaking after 24 h then decreasing; and a second wave that increases over time until disease resolution or death (111) (Figure 2). In the ferret model, we have shown that the neutrophils become concentrated at specific foci in the lungs coincident with influenza-positive epithelium and the expression of chemoattractant chemokine genes (22). Neutrophil chemotaxis in humans is thought to be mediated by many factors, such as the chemokine CXCL8, cytokines IL-1 and TNFα, and complement C5a (145, 147, 148). During both mild and severe IAV disease, patients show increased blood CRP and activation of C5a (149), as well as increased secretion of CXCL8, TNFα, and IL-1 in nasal washes, which correlate with disease severity (87, 89, 93, 150–152). In the mouse model of influenza virus infection, chemical reduction of C5a during IAV infection reduced lung neutrophilia (153). Similarly, knockout mice deficient in the inflammasome pathway or mice not expressing cytokines, such as IL-1b and IL-6, have decreased neutrophil activation and migration to the lungs during IAV infection (Table S1 in Supplementary Material) (154–156). Mice do not possess CXCL8, but CXCL1 and CXCL2 have equivalent functions. Neutrophils contribute CXCL2 to the IAV-infected mouse lung to further stimulate neutrophil recruitment (157). More recently, it was shown that removing a CXCL1 repressor (*Setdb2*) does not increase recruitment of neutrophils to the lungs of mice infected with IAV PR8, rather it reduces the ability to respond to bacterial superinfection (158). In addition, it was shown that another ISG, CXCL10, operates on a unique subset of CXCR3+ neutrophils present during mouse IAV infection in an autocrine manner, increasing chemotaxis, oxidative burst, and enhancing inflammation (157) (Tables S3 and S4 in Supplementary Material). Finally, aryl hydrocarbon receptor is somehow linked to increases in NO and neutrophilia in the lungs of IAV-infected mice independently of known neutrophil chemoattractants or mechanisms of neutrophil extravasation (159–162).

### Neutrophil Extracellular PRRs and Phagocytosis

In cell coculture, human neutrophils were seen to interact specifically with IAV-infected cells (163), although the nature of this interaction in the infected lung is unknown. Neutrophils are phagocytic cells, and their methods for sensing extracellular pathogens rely on TLRs (2, 147). Stimulation of neutrophils through cell surface TLRs has been recorded to promote cytokine secretion (CXCL8 and TNFα *via* NFκB and AP-1), formation of reactive oxygen species (ROS), phagocytosis, granule secretion, neutrophil extracellular trap (NET) formation, and migration (145, 147, 148). Human neutrophils highly express nucleic acid-detecting TLRs, specifically endosomal TLR8 (164, 165), but do not express nor respond to activators of TLR3 or TLR7 (164–166). [Interestingly, TLR3^−/−^ mice have increased neutrophilia and fewer macrophages in the lungs, yet have increased survival after infection with IAV (167, 168).] TLR4 is required for LPS-induced neutrophil migration to the lung (169) and can stimulate immunostimulatory responses *via* TRIF adaptors (170); however, TLR4-stimulation does not lead to the production of type I IFN in neutrophils (166, 171).

Several innate immune effector proteins with opsonizing functions that are present in airway mucosae are known to interact with both IAV and neutrophils. Surfactant protein D, a lung collectin, is an innate immune defense against a variety of viruses, opsonizing the viruses for phagocytosis by neutrophils, which in turn causes the production of ROS (172–178). Human neutrophil defensins are short basic peptides released from neutrophil granules during inflammation (145, 178, 179). They have been shown to interact with IAV, reducing infectivity, and promote neutrophil phagocytosis and clearance of IAV (178–182). Defensins may also buffer the oxidative burst from neutrophils that follows from phagocytosis of viruses that have been opsonized by surfactant protein D (178–180). One study indicated that neutrophils do not interact with immunoglobulin-bound IAV (183); however, another showed that protective anti-IAV antibody therapy only protected mice in the presence of neutrophils (184). The hypothesis that IAV pathogenicity can be partially explained by infection with viral variants that can evade opsonization by innate immune effectors is attractive and deserves further study (176).

### Neutrophil Intracellular PRRs

Neutrophils express sialic acid receptors and may become infected with IAV (74, 185, 186). Neutrophils infected with IAV have increased apoptosis, but infection does not result in the production of virus (186). Infection of human neutrophils with IAV treated at 56°C to denature the viral replicase but not HA suggested that infection alone, but not replication, is sufficient to stimulate the release of CXCL8 and CCL4 in human neutrophils (187). Neutrophils infected with IAV have rapid upregulation (<9 hpi) of type I IFN pathways, including cytoplasmic PRRs, IFNβ, and ISGs (186), which is counter to the long-held dogma that neutrophils were incapable of gene expression. This is may be due to RIG-like receptors (RLRs) sensing of viral dsRNA, as neutrophils transfected with poly(I:C) (a viral RNA mimic) have a similar response (166). Additionally, neutrophils express nod-like receptors but it is unclear how these interact with IAV infection (188, 189). In general, inflammasome and pro-IL1 activation following IAV infection is poorly understood (154, 155, 190, 191). However, studies of IAV infection using caspase-1, IL-1β, or IL-1R transgenic mice show modulation of neutrophil infiltration and pathology and suggest that it is an IAV subtype-dependent effect (154, 191–195) (Tables S1 and S2 in Supplementary Material). Furthermore, it has been demonstrated that the HA of some IAV isolates suppresses neutrophil activation, providing further evidence for IAV subtype-dependent effects on neutrophils (185, 186, 196).

### Neutrophil Activation and Degranulation

Activation of TLRs and RLRs trigger degranulation and the expression of surface CD11b (166), which pairs with CD18 to form the “Mac-1” integrin dimer that binds collagen (197). This facilitates migration through tissues, and release of gelatinase or collagenase (MMP-2 and MMP-9) from neutrophils assist in clearing connective tissue from the path. At the site of infection, neutrophils release microbial effectors [reviewed in Ref. (145, 198)]. Neutrophils develop granules sequentially (azurophilic, specific, gelatinase, secretory) and secrete granules in the reverse order (145). Secretory and gelatinase granules are released shortly after endothelial transmigration and contain membrane proteins essential for movement [extracellular matrix (ECM)-binding integrins] and pathogen recognition [immunoglobulin (FcR) and complement receptors] (145). Specific and azurophilic granules contain tissue-destroying enzymes and antimicrobial proteins. For example, neutrophil myeloperoxidase (MPO) may contribute to lung injury during IAV infection (199); however, it may have direct antiviral effects on IAV (200). An investigation found no difference between IAV infection of a wild-type and neutrophil elastase knockout mouse, measuring lung function, chemokine secretion, and neutrophil recruitment (201) (Table S5 in Supplementary Material). The contents of the granules can be secreted to destroy ECM (such as MMP-9) or directed toward phagosomes to destroy engulfed microbes. The production of hypochlorous acid (HOCl) is the main oxidant used in phagosomal killing, and its production is dependent on the generation of ROS by the neutrophils. Interestingly, infection by IAV was related to the inhibition of phagosomal killing of bacteria (196).

Neutrophil degranulation primes neutrophils for ROS generation by mobilizing NADPH oxidase components to the plasma membrane (145) and exocytosis of MPO. IAV infection causes the generation of ROS in neutrophils (202). Oxidative burst is thought to have direct microbial effects; however, the direct effect on IAV has not been published (203). IAV infection benefits from the presence of ROS in the environment (204, 205), yet IAV also suppresses NADPH oxidase activity within infected phagocytes (206, 207). Many have investigated the effects of ROS and NO on lung inflammation during IAV infection and found that reduction of oxidative stress in the form of both ROS and NO alieves IAV-dependent lung injury (206, 208–213) (Table S5 in Supplementary Material). For example, oxidized lipids in the lung environment may trigger TLR4, activating immune cells and contributing to increased lung injury (214–216).

### Neutrophil Netosis

Neutrophils undergo a form of programmed cell death called netosis, in which NETs are formed (217). NETs are extracellular strands DNA wrapped in histones and enriched in neutrophil effector proteins (e.g., neutrophil elastase and MPO) (218). NETs have the effect of killing many pathogens, including bacteria (146), fungi (219), protozoans (220), and more recently viruses (221). NETs are becoming the focus of study in autoimmune disease atherosclerosis, since they damage endothelium (222–224). Recently, it was shown that hantavirus stimulates NET production during infection, which leads to the generation of autoantibodies and may provide a mechanism for the hemorrhagic fever caused by Old World hantaviruses (225). NETs are typically found with histones, MPO, and neutrophil elastase, and the effect is to isolate the effects of these molecules directly onto the pathogen surface with a “sticky” NET of nucleic acid. NETs contribute to acute lung injury and alveolar capillary damage during IAV infection (139). Yet, very little is known about the relationship between NETs and viral infection in viral disease pathogenesis (226).

In summary, neutrophils are capable of recognizing viruses *via* PRRs as either opsonized virions or *via* endosomal TLR. Although the signaling cascade differs from other phagocytic cells, neutrophils are capable of responding to viral PAMPs with respiratory burst, degranulation of proteases and cytokines, and/or netosis. It is not clear if these responses are effective against influenza virus; in fact, evidence exists that suggest influenza viruses may take advantage of the inflammatory environment. More importantly, it is not clear if there are differences in response to different influenza virus subtypes or strains. As is true for many respiratory etiologies, neutrophil responses must be balanced during influenza virus infection to adequately control of inflammation while promoting pro-immune responses. The timing of neutrophils during disease progression correlates with a key point in divergent disease outcomes (Figure 2), and neutrophils may act both globably and locally at foci of infection (22, 111). Thus, neutrophils are focused in the airways at critical timepoints following infection and therefore balancing their potent inflammatory effector functions may determine disease outcome.

## Comparisons of Neutrophils in Viral Respiratory Diseases

As discussed above, there appears to be a correlation between the timing and location of IAV infection and the action of neutrophils, but evidence directly linking these phenomena together remains overall circumstantial. In contrast, during bacterial pneumonia there is direct evidence of the importance of neutrophils in disease: bacterial PAMPs directly upregulate neutrophil activating and chemoattractant chemokines, bacteria have defined anti-neutrophil functions, and some bacteria, e.g., *Mycobacterium tuberculosis*, rely on neutrophils to establish their granulomatous niche (6, 227, 228). Similarly, we suggest that viruses may interact with respiratory cells to create a viral microenvironmental niche. To further substantiate a link between neutrophils and virus infection, evidence for the role of neutrophils in selected viral respiratory diseases is summarized in Tables 1 and 2. Discussion below focuses on common patterns of neutrophil responses in severe and mild forms of respiratory viral infection.

**Table 1:** **(−) Sense RNA respiratory viruses that cause increased neutrophil infiltration during infection**.

Virus family	Virus type	Host/model	Primary airway target cell	Pathology	Neutrophil abundance	Reference
Orthomyxoviridae	Influenza A virus	Hu, NHP, Mo, Fe, Sw	Epithelial cells [upper respiratory tract (URT), lower respiratory tract (LRT)]	Mild: necrotic rhinitis and tracheitis; moderate: necrotic bronchiolitis and alveolitis; severe: diffuse alveolar damage and hypercytokinemia	(+++)	(229–231)
Paramyxoviridae	Human respiratory syncytial virus	Hu	Epithelial cells (URT, LRT)	Severe: necrotic bronchiolitis and alveolitis, obstructed bronchioles, and giant cell formation	(+++)	(232, 233)
Paramyxoviridae	Human metapneumovirus	Hu	Epithelium	Mild: airway inflammation and epithelial degeneration	(+)	(234, 235)
Paramyxoviridae	Hendrah/Nipah virus	Hu	Not defined	Severe: interstitial pneumonia, but primarily vasotropic or neurotropic	(++)	(236)
Paramyxoviridae	Measles virus	Hu, Mo	Resident myeloid cells of the lung	Severe: bronchiolitis obliterans	(++)	(237)
Paramyxoviridae	Human parainfluenza virus	Hu	Ciliated epithelium	Bronchiolitis and alveolitis	Not defined	(238)
Bunyaviridae	New World hantavirus	Hu	Lung microvascular endothelium	Hantavirus pulmonary syndrome, endothelial infilammation, and focal antigen-positive sites in lung	(+)	(239)

*Hu, human; NHP, non-human primate; Mo, mouse; Fe; ferret; Sw, swine*.

**Table 2:** **(+) Sense RNA and DNA respiratory viruses that cause increased neutrophil infiltration during infection**.

Virus family	Virus type	Host/model	Primary cell target	Pathology	Neutrophil abundance	Reference
Picornaviridae (+RNA)	Human rhinovirus	Hu	Epithelium	Mild to moderate: neutrophilic rhinitis; severe: acute LRT, bronchiolitis, and alveolitis	(+++)	(231)
Adenoviridae (dsDNA)	Human adenovirus (HAdv3, HAdv7)	Hu	Epithelium	Bronchitis and alveolitis	(+)	(240)
Coronaviridae (+RNA)	Human coronavirus (NL-63 or OC43)	Hu	Epithelium	Mild	(+/−)	(241)
Coronaviridae (+RNA)	Severe acute respiratory syndrome coronavirus	Hu, Fe	Epithelium	Alveolitis, acute respiratory distress syndrome (ARDS), hypercytokinemia	(++)	(10)
Coronaviridae (+RNA)	Middle east respiratory syndrome coronavirus	Hu, Fe	Epithelium	Alveolitis, ARDS, hypercytokinemia	(++)	(11)

*Hu, humans; Fe, ferrets*.

### Severe Viral Respiratory Disease

For both viral and bacterial etiologies, the most severe clinical complications result from infection of the LRT. Infection of the LRT by viruses, such as human parainfluenza viruses, influenza A(H1N1)pdm09, HPAI, New World hantavirus infections (causing hantavirus pulmonary syndrome), *Severe acute respiratory syndrome-related coronavirus* (SARS-Cov), and Middle East respiratory syndrome coronavirus (MERS-CoV), are all associated with neutrophilic infiltration at sites of infection to various degrees and may develop into ARDS (9–11, 19, 60, 131, 242–245). Clinically defined, ARDS has three phases; and most patents die within the first phase, the acute or “exudative” phase [reviewed in Ref. (12–14)]. This phase is characterized by an increased immune response with high production of pro-inflammatory cytokines and chemokines, increased neutrophil infiltration and accumulation in the alveoli, and disruption of the alveolar epithelial–capillary barrier, which leads to increased vascular permeability and edema (13). The distinctive role for lung neutrophil infiltration in viral infection is summarized as follows: some, but not all, viruses that infect the LRT result in clinically defined ARDS, and lung neutrophil infiltration is associated with viruses that do and do not lead to ARDS (3, 5, 9, 10, 60, 131, 199, 242–244, 246, 247). In ARDS, there are data that directly support the role of neutrophils as both beneficial and detrimental (3, 13, 14). Perhaps, there are general factors (host or virus) that lead to a common antiviral response of neutrophils.

### Mild Viral Respiratory Disease

In contrast to emergent highly pathogenic respiratory viruses, notable “mild” human respiratory viruses also involve increased neutrophils at the site of infection (e.g., hRSV). As expected, infection with these viruses is typically associated with the increase of neutrophil chemoattractant chemokines. For example, infection with HRV is a well-studied virus for which there are several studies on the link between neutrophils and disease (229–231, 248) (Table 2). HRV virions enter nasal epithelial cells *via* endocytosis, yet, unlike influenza, infection does not cause major damage to the nasal epithelium (249–251). Neutrophilic rhinitis, increased vascular permeability, and mucus hypersecretion are the key pathological features of HRV infection (229–231, 249), and infected epithelium seems to be the source of large amounts of neutrophil chemotactic molecules, particularly CXCL8 and kinins (229, 248, 252, 253). Interestingly, *in vitro* studies have shown that viral recognition of HRV shares features with hRSV, but is somewhat different than with IAV (254–256). It has been established that there are virus-specific and cell-specific differences in sensing RNA viruses *via* primarily TLR- and/or RLR-pathways (and even in a preference for RIG-I versus MDA5), yet these pathways may have similar general endpoints, such as chemokine and cytokine signaling (255, 257–259).

Finally, a key question is whether virus-induced cytopathy drives neutrophilia or whether it is the result of host response to viral infection. Infections with influenza A(H1N1)pdm09 virus, HPAI, SARS-CoV, and MERS-CoV are thought to cause acute lung injury which results in ARDS; characterized by excessive damage to the alveolar epithelium and involving the infiltration of neutrophils (67, 68) (Tables 1 and 2). However, less pathogenic strains such as HRV infections do not cause significant damage to the respiratory mucosa, yet neutrophils are present (249, 250). Conversely, viruses that cause moderate, focal cytopathy in the lungs, for example seasonal IAV and hRSV, are known to cause neutrophilic infiltration (250, 252, 253). Therefore, neutrophils are not necessarily associated with direct cytopathic effect nor are they exclusively associated with severe disease.

### Bacterial Respiratory Disease

Despite many years of searching, there is no single reliable biomarker to indicate a bacterial versus viral infection (although CRP is a good candidate). This is surprising, given the otherwise significant fundamental differences between the biology of these two types of pathogens; differences which are reflected in general immune responses and begin with pathogen detection. Signaling through TLR on the plasma membrane versus endosomal or cytoplasmic PRRs is controlled by complex intracellular adaptor proteins [reviewed in Ref. (260)]. For example, the complexities of signaling allow the characteristically bacteria-specific TLR4 to signal the upregulation of immunostimulatory type I IFN characteristic of a virus infection (170). As evidenced above, many viral infections associated with neutrophil infiltration have RNA genomes. Host cells detect RNA viruses primarily through RLR as well as TLR, whereas bacteria rely on a different group of PRRs to detect extracellular PAMPs (255, 261–263). Interestingly, the lung microenvironment to hRSV has been shown to be different from IAV, specifically in the presence of IL-4 (264, 265). It is thought that this is driven by the presence of alternatively activated macrophages during RSV infection (266). It is not known if this directs differences in neutrophil chemoattraction, yet IL-4 is known to drive a Th2 (“bacterial” or antibody-biased) immune response (267). In sum, surprisingly little is clinically different between the innate immune responses to viral versus bacterial infection; however, perhaps comparative studies that focus on neutrophils can uncover virus-specific responses.

## Neutrophils in the Viral Microenvironment

In general, immune activation pathways that involve the activation of NFκB lead to the secretion of neutrophil chemotactic chemokines [reviewed in Ref. (268)]. This is heavily driven by PAMP recognition and activation pathways, and during a viral infection type I IFNs and ISGs are the unique elements in the virus-inflamed lung environment [reviewed in Ref. (24–26, 269, 270)]. This single difference between viral and bacterial infections could have drastic effects on the actions of neutrophils once in the lung. Moreover, without this information (e.g., pathogenic viruses that suppress type I IFN) neutrophils may respond to inflammation in an inefficient way, potentially with pathologic effects (23, 186, 256, 271–277). Neutrophils are known to respond to IAV and type I IFN by upregulation of ISGs (186). In systemic lupus erythematosus, neutrophils may be a large contributor of type I IFN (278, 279). During increased inflammation, left-shifted or immature neutrophils emerge from the bone marrow—a classic sign for sepsis, but also known to be present during some severe viral respiratory diseases (e.g., HPS) (239). It is known that immature neutrophils do neither express IFNα/β receptor nor many other cytokine receptors (273). It is unknown what affect this would have during increased inflammation during pathogenic influenza infection, although their role in other inflammatory conditions suggests it may affect their functions (271, 273, 280, 281).

### Resolving Inflammation

All forms of respiratory infection require resolution of the infection and inflammation. IFNs are essential components of initiating sterilizing immunity to virus infection *via* the adaptive immune system (i.e., resolution of infection), at which point the resolution of inflammation can effectively proceed (25, 26, 282). Although the mechanisms are poorly understood, through their direct antiviral actions and indirect actions on the lung microenvironment (e.g., efferocytosis of apoptotic neutrophils by macrophages), neutrophils have the ability to influence outcomes toward successful resolution as well as toward the formation of ARDS (3, 5, 12, 13, 21, 74, 166, 184, 198, 199, 283–285). Thus, there is evidence that the role of neutrophils in viral infections of the respiratory system is not limited to inflammation, but likely includes recovery from infection and the initiation of adaptive immunity (74, 138, 284). Apart from initiating adaptive immunity, resolution of inflammation may be partially regulated by secretion of IL-1RA and chemokine-destroying factors by recruited macrophages (286, 287). Additionally, it has been proposed that efferocytosis of apoptotic neutrophils is a key step in resolution of inflammation (288–290), and occurs in the lung during bacterial pneumonia (291). It is unclear if this happens during IAV infection or infection with other respiratory viruses.

### Prolonging Inflammation

Factors that prolong the life span of neutrophils in the lungs increase the probability that they may contribute to immunopathology. IL-6 and G-CSF are immune mediators present in the lung during infection and are known to prolong survival of neutrophils in mouse lungs following IAV infection (292). Both neutrophils and macrophages are known to phagocytose apoptotic epithelial cells in mouse lungs during IAV infection (283). The cells may be recruited *via* chemokines or damage receptors. For example, necrotic IAV-infected epithelial cells are a source of CXCL8 (293), which attract neutrophils to dying cells. In addition, neutrophils can detect DAMPs such as S100A9 (294). It has been shown that extracellular S100A9 is abundant during IAV infection in mice (295). Antibody-mediated neutralization of S100A9 decreased lung inflammation in mice and improved disease outcome (295). Apart from potential tissue-destroying effects of neutrophil proteases, the presence of NETs may induce even more inflammation in the lungs (21, 139). Thus, there are limited data supporting directly malevolent actions of neutrophils (Table S5 in Supplementary Material), yet factors that increase their presence and prolong their survival in the lung are correlated with increased disease severity.

## Concluding Remarks

There are substantial data that suggest neutrophils are a part of a viral response to infection. Neutrophils are among the first responders to IAV infection in the lung, and they remain in great numbers throughout the development of ARDS. Although neutrophils are an important component of the general response to infection in the respiratory system, as is discussed herein, neutrophils are capable of recognizing viruses (*via* viral PAMPs), responding to viruses with specific effector functions, and may be instrumental in determining disease outcome. Evidence exists to support the hypothesis that neutrophils respond specifically to the focal nature of viral infection, and they act to influence this microenvironment *via* their virus-specific effector functions. Factors that influence successful recovery from respiratory viral infection (versus lethal outcome) are complex and both host- and virus-specific. However, a better understanding of the role neutrophils, previously underappreciated with respect to viral infections, will reveal important information about disease outcome. Many questions remain before it is determined the part neutrophils play in mild and severe disease.

## Author Contributions

Both authors contributed to the formulation of this scientific research topic. JC wrote the manuscript as part of his Ph.D. dissertation under the mentorship of CJ (296).

## Conflict of Interest Statement

The authors declare that the research was conducted in the absence of any commercial or financial relationships that could be construed as a potential conflict of interest.

## References

[1] Cowland JB Borregaard N. Granulopoiesis and granules of human neutrophils. Immunol Rev (2016) 273:11–28. 10.1111/imr.12440 27558325

[2] Galani IE Andreakos E. Neutrophils in viral infections: current concepts and caveats. J Leukoc Biol (2015) 98:557–64. 10.1189/jlb.4VMR1114-555R 26160849

[3] Abraham E. Neutrophils and acute lung injury. Crit Care Med (2003) 31:S195–9. 10.1097/01.CCM.0000057843.47705.E8 12682440

[4] Burns AR Smith CW Walker DC. Unique structural features that influence neutrophil emigration into the lung. Physiol Rev (2003) 83:309–36. 10.1152/physrev.00023.2002 12663861

[5] Grommes J Soehnlein O. Contribution of neutrophils to acute lung injury. Mol Med (2011) 17:293–307. 10.2119/molmed.2010.00138 21046059 PMC3060975

[6] Lowe DM Redford PS Wilkinson RJ O’Garra A Martineau AR. Neutrophils in tuberculosis: friend or foe? Trends Immunol (2012) 33:14–25. 10.1016/j.it.2011.10.003 22094048

[7] McDonald B Kubes P. Cellular and molecular choreography of neutrophil recruitment to sites of sterile inflammation. J Mol Med (Berl) (2011) 89:1079–88. 10.1007/s00109-011-0784-9 21751029

[8] Smith PK Wang SZ Dowling KD Forsyth KD. Leucocyte populations in respiratory syncytial virus-induced bronchiolitis. J Paediatr Child Health (2001) 37:146–51. 10.1046/j.1440-1754.2001.00618.x 11328469

[9] Nicholls JM Poon LL Lee KC Ng WF Lai ST Leung CY Lung pathology of fatal severe acute respiratory syndrome. Lancet (2003) 361:1773–8. 10.1016/S0140-6736(03)13413-7 12781536 PMC7112492

[10] Tse GM To KF Chan PK Lo AW Ng KC Wu A Pulmonary pathological features in coronavirus associated severe acute respiratory syndrome (SARS). J Clin Pathol (2004) 57:260–5. 10.1136/jcp.2003.013276 14990596 PMC1770245

[11] van den Brand JM Smits SL Haagmans BL. Pathogenesis of Middle East respiratory syndrome coronavirus. J Pathol (2015) 235:175–84. 10.1002/path.4458 25294366 PMC7167882

[12] Matthay MA Ware LB Zimmerman GA. The acute respiratory distress syndrome. J Clin Invest (2012) 122:2731–40. 10.1172/JCI60331 22850883 PMC3408735

[13] Ware LB Matthay MA The acute respiratory distress syndrome. N Engl J Med (2000) 342:1334–49. 10.1056/NEJM200005043421806 10793167

[14] Abraham E Matthay MA Dinarello CA Vincent JL Cohen J Opal SM Consensus conference definitions for sepsis, septic shock, acute lung injury, and acute respiratory distress syndrome: time for a reevaluation. Crit Care Med (2000) 28:232–5. 10.1097/00003246-200001000-00039 10667529

[15] Aherne W Bird T Court SD Gardner PS McQuillin J. Pathological changes in virus infections of the lower respiratory tract in children. J Clin Pathol (1970) 23:7–18. 10.1136/jcp.23.1.7 4909103 PMC474401

[16] Craig A Mai J Cai S Jeyaseelan S Neutrophil recruitment to the lungs during bacterial pneumonia. Infect Immun (2009) 77:568–75. 10.1128/IAI.00832-08 19015252 PMC2632043

[17] Herold S Mayer K Lohmeyer J. Acute lung injury: how macrophages orchestrate resolution of inflammation and tissue repair. Front Immunol (2011) 2:65. 10.3389/fimmu.2011.00065 22566854 PMC3342347

[18] Lien DC Wagner WW Jr Capen RL Haslett C Hanson WL Hofmeister SE Physiological neutrophil sequestration in the lung: visual evidence for localization in capillaries. J Appl Physiol (1985) (1987) 62:1236–43. 3106311 10.1152/jappl.1987.62.3.1236

[19] Rubenfeld GD Caldwell E Peabody E Weaver J Martin DP Neff M Incidence and outcomes of acute lung injury. N Engl J Med (2005) 353:1685–93. 10.1056/NEJMoa050333 16236739

[20] Reutershan J Basit A Galkina EV Ley K. Sequential recruitment of neutrophils into lung and bronchoalveolar lavage fluid in LPS-induced acute lung injury. Am J Physiol Lung Cell Mol Physiol (2005) 289:L807–15. 10.1152/ajplung.00477.2004 15951336

[21] Jenne CN Wong CH Zemp FJ McDonald B Rahman MM Forsyth PA Neutrophils recruited to sites of infection protect from virus challenge by releasing neutrophil extracellular traps. Cell Host Microbe (2013) 13:169–80. 10.1016/j.chom.2013.01.005 23414757

[22] Camp JV Bagci U Chu YK Squier B Fraig M Uriarte SM Lower respiratory tract infection of the ferret by 2009 H1N1 pandemic influenza A virus triggers biphasic, systemic, and local recruitment of neutrophils. J Virol (2015) 89:8733–48. 10.1128/JVI.00817-15 26063430 PMC4524093

[23] Davidson S Crotta S McCabe TM Wack A Pathogenic potential of interferon alphabeta in acute influenza infection. Nat Commun (2014) 5:3864 10.1038/ncomms4864 24844667 PMC4033792

[24] Garcia-Sastre A Biron CA Type 1 interferons and the virus-host relationship: a lesson in detente. Science (2006) 312:879–82. 10.1126/science.1125676 16690858

[25] Le Bon A Tough DF. Links between innate and adaptive immunity via type I interferon. Curr Opin Immunol (2002) 14:432–6. 10.1016/S0952-7915(02)00354-0 12088676

[26] Stetson DB Medzhitov R. Type I interferons in host defense. Immunity (2006) 25:373–81. 10.1016/j.immuni.2006.08.007 16979569

[27] Tisoncik JR Korth MJ Simmons CP Farrar J Martin TR Katze MG. Into the eye of the cytokine storm. Microbiol Mol Biol Rev (2012) 76:16–32. 10.1128/MMBR.05015-11 22390970 PMC3294426

[28] Watanabe T Tisoncik-Go J Tchitchek N Watanabe S Benecke AG Katze MG 1918 Influenza virus hemagglutinin (HA) and the viral RNA polymerase complex enhance viral pathogenicity, but only HA induces aberrant host responses in mice. J Virol (2013) 87:5239–54. 10.1128/JVI.02753-12 23449804 PMC3624330

[29] Vidy A Maisonnasse P Da Costa B Delmas B Chevalier C Le Goffic R. The influenza virus protein PB1-F2 increases viral pathogenesis through neutrophil recruitment and NK cells inhibition. PLoS One (2016) 11:e0165361. 10.1371/journal.pone.0165361 27798704 PMC5087861

[30] Webster RG Bean WJ Gorman OT Chambers TM Kawaoka Y. Evolution and ecology of influenza A viruses. Microbiol Rev (1992) 56:152–79. 1579108 10.1128/mr.56.1.152-179.1992 PMC372859

[31] Webster RG. Predictions for future human influenza pandemics. J Infect Dis (1997) 176(Suppl 1):S14–9. 10.1086/514168 9240688

[32] Webster RG. Influenza virus: transmission between species and relevance to emergence of the next human pandemic. Arch Virol Suppl (1997) 13:105–13. 9413531 10.1007/978-3-7091-6534-8_11

[33] Webster RG Shortridge KF Kawaoka Y Influenza: interspecies transmission and emergence of new pandemics. FEMS Immunol Med Microbiol (1997) 18:275–9. 10.1111/j.1574-695X.1997.tb01056.x 9348163 PMC7314015

[34] Webster RG Influenza: an emerging disease. Emerg Infect Dis (1998) 4:436–41. 10.3201/eid0403.980325 9716966 PMC2640312

[35] Cox NJ Subbarao K. Global epidemiology of influenza: past and present. Annu Rev Med (2000) 51:407–21. 10.1146/annurev.med.51.1.407 10774473

[36] CDC. Outbreak of swine-origin influenza A (H1N1) virus infection – Mexico, March-April 2009. MMWR Morb Mortal Wkly Rep (2009) 58:467–70. 19444150

[37] Cao B Li XW Mao Y Wang J Lu HZ Chen YS Clinical features of the initial cases of 2009 pandemic influenza A (H1N1) virus infection in China. N Engl J Med (2009) 361:2507–17. 10.1056/NEJMoa0906612 20007555

[38] Donaldson LJ Rutter PD Ellis BM Greaves FE Mytton OT Pebody RG Mortality from pandemic A/H1N1 2009 influenza in England: public health surveillance study. BMJ (2009) 339:b5213. 10.1136/bmj.b5213 20007665 PMC2791802

[39] Garten RJ Davis CT Russell CA Shu B Lindstrom S Balish A Antigenic and genetic characteristics of swine-origin 2009 A(H1N1) influenza viruses circulating in humans. Science (2009) 325:197–201. 10.1126/science.1176225 19465683 PMC3250984

[40] Jain S Kamimoto L Bramley AM Schmitz AM Benoit SR Louie J Hospitalized patients with 2009 H1N1 influenza in the United States, April-June 2009. N Engl J Med (2009) 361:1935–44. 10.1056/NEJMoa0906695 19815859

[41] Smith GJ Vijaykrishna D Bahl J Lycett SJ Worobey M Pybus OG Origins and evolutionary genomics of the 2009 swine-origin H1N1 influenza A epidemic. Nature (2009) 459:1122–5. 10.1038/nature08182 19516283

[42] Gill JR Sheng ZM Ely SF Guinee DG Beasley MB Suh J Pulmonary pathologic findings of fatal 2009 pandemic influenza A/H1N1 viral infections. Arch Pathol Lab Med (2010) 134:235–43. 10.1043/1543-2165-134.2.235 20121613 PMC2819217

[43] Libster R Bugna J Coviello S Hijano DR Dunaiewsky M Reynoso N Pediatric hospitalizations associated with 2009 pandemic influenza A (H1N1) in Argentina. N Engl J Med (2010) 362:45–55. 10.1056/NEJMoa0907673 20032320

[44] Agarwal PP Cinti S Kazerooni EA. Chest radiographic and CT findings in novel swine-origin influenza A (H1N1) virus (S-OIV) infection. AJR Am J Roentgenol (2009) 193:1488–93. 10.2214/AJR.09.3599 19933638

[45] Mollura DJ Asnis DS Crupi RS Conetta R Feigin DS Bray M Imaging findings in a fatal case of pandemic swine-origin influenza A (H1N1). AJR Am J Roentgenol (2009) 193:1500–3. 10.2214/AJR.09.3365 19933640 PMC2788497

[46] Neumann G Noda T Kawaoka Y. Emergence and pandemic potential of swine-origin H1N1 influenza virus. Nature (2009) 459:931–9. 10.1038/nature08157 19525932 PMC2873852

[47] Peiris JS Poon LL Guan Y. Emergence of a novel swine-origin influenza A virus (S-OIV) H1N1 virus in humans. J Clin Virol (2009) 45:169–73. 10.1016/j.jcv.2009.06.006 19540800 PMC4894826

[48] Perez-Padilla R de la Rosa-Zamboni D Ponce de Leon S Hernandez M Quinones-Falconi F Bautista E Pneumonia and respiratory failure from swine-origin influenza A (H1N1) in Mexico. N Engl J Med (2009) 361:680–9. 10.1056/NEJMoa0904252 19564631

[49] Woo PC Tung ET Chan KH Lau CC Lau SK Yuen KY. Cytokine profiles induced by the novel swine-origin influenza A/H1N1 virus: implications for treatment strategies. J Infect Dis (2010) 201:346–53. 10.1086/649785 20030555 PMC7202468

[50] Calore EE Uip DE Perez NM. Pathology of the swine-origin influenza A (H1N1) flu. Pathol Res Pract (2011) 207:86–90. 10.1016/j.prp.2010.11.003 21176866

[51] Shieh WJ Blau DM Denison AM Deleon-Carnes M Adem P Bhatnagar J 2009 pandemic influenza A (H1N1): pathology and pathogenesis of 100 fatal cases in the United States. Am J Pathol (2010) 177:166–75. 10.2353/ajpath.2010.100115 20508031 PMC2893660

[52] Fukuyama S Kawaoka Y. The pathogenesis of influenza virus infections: the contributions of virus and host factors. Curr Opin Immunol (2011) 23:481–6. 10.1016/j.coi.2011.07.016 21840185 PMC3163725

[53] Medina RA Garcia-Sastre A. Influenza A viruses: new research developments. Nat Rev Microbiol (2011) 9:590–603. 10.1038/nrmicro2613 21747392 PMC10433403

[54] Tscherne DM Garcia-Sastre A. Virulence determinants of pandemic influenza viruses. J Clin Invest (2011) 121:6–13. 10.1172/JCI44947 21206092 PMC3007163

[55] Belser JA Katz JM Tumpey TM. The ferret as a model organism to study influenza A virus infection. Dis Model Mech (2011) 4(5):575–9. 10.1242/dmm.007823 21810904 PMC3180220

[56] Smith H Sweet C. Lessons for human influenza from pathogenicity studies with ferrets. Rev Infect Dis (1988) 10:56–75. 10.1093/clinids/10.1.56 3281223

[57] Barnard DL. Animal models for the study of influenza pathogenesis and therapy. Antiviral Res (2009) 82:A110–22. 10.1016/j.antiviral.2008.12.014 19176218 PMC2700745

[58] Belser JA Szretter KJ Katz JM Tumpey TM. Use of animal models to understand the pandemic potential of highly pathogenic avian influenza viruses. Adv Virus Res (2009) 73:55–97. 10.1016/S0065-3527(09)73002-7 19695381

[59] de Jong MD Simmons CP Thanh TT Hien VM Smith GJ Chau TN Fatal outcome of human influenza A (H5N1) is associated with high viral load and hypercytokinemia. Nat Med (2006) 12:1203–7. 10.1038/nm1477 16964257 PMC4333202

[60] van den Brand JM Haagmans BL van Riel D Osterhaus AD Kuiken T. The pathology and pathogenesis of experimental severe acute respiratory syndrome and influenza in animal models. J Comp Pathol (2014) 151:83–112. 10.1016/j.jcpa.2014.01.004 24581932 PMC7094469

[61] van Riel D Munster VJ de Wit E Rimmelzwaan GF Fouchier RA Osterhaus AD Human and avian influenza viruses target different cells in the lower respiratory tract of humans and other mammals. Am J Pathol (2007) 171:1215–23. 10.2353/ajpath.2007.070248 17717141 PMC1988871

[62] Watanabe T Kawaoka Y Pathogenesis of the 1918 pandemic influenza virus. PLoS Pathog (2011) 7:e1001218 10.1371/journal.ppat.1001218 21298032 PMC3029258

[63] Cameron CM Cameron MJ Bermejo-Martin JF Ran L Xu L Turner PV Gene expression analysis of host innate immune responses during lethal H5N1 infection in ferrets. J Virol (2008) 82:11308–17. 10.1128/JVI.00691-08 18684821 PMC2573250

[64] Lu X Tumpey TM Morken T Zaki SR Cox NJ Katz JM. A mouse model for the evaluation of pathogenesis and immunity to influenza A (H5N1) viruses isolated from humans. J Virol (1999) 73:5903–11. 10364342 10.1128/jvi.73.7.5903-5911.1999 PMC112651

[65] To KF Chan PK Chan KF Lee WK Lam WY Wong KF Pathology of fatal human infection associated with avian influenza A H5N1 virus. J Med Virol (2001) 63:242–6. 10.1002/1096-9071(200103)63:3<242::AID-JMV1007>3.0.CO;2-N 11170064

[66] Zitzow LA Rowe T Morken T Shieh WJ Zaki S Katz JM. Pathogenesis of avian influenza A (H5N1) viruses in ferrets. J Virol (2002) 76:4420–9. 10.1128/JVI.76.9.4420-4429.2002 11932409 PMC155091

[67] Kuiken T Taubenberger JK. Pathology of human influenza revisited. Vaccine (2008) 26(Suppl 4):D59–66. 10.1016/j.vaccine.2008.07.025 19230162 PMC2605683

[68] Taubenberger JK Morens DM. The pathology of influenza virus infections. Annu Rev Pathol (2008) 3:499–522. 10.1146/annurev.pathmechdis.3.121806.154316 18039138 PMC2504709

[69] Peiris JS Yu WC Leung CW Cheung CY Ng WF Nicholls JM Re-emergence of fatal human influenza A subtype H5N1 disease. Lancet (2004) 363:617–9. 10.1016/S0140-6736(04)15595-5 14987888 PMC7112424

[70] Yuen KY Chan PK Peiris M Tsang DN Que TL Shortridge KF Clinical features and rapid viral diagnosis of human disease associated with avian influenza A H5N1 virus. Lancet (1998) 351:467–71. 10.1016/S0140-6736(98)01182-9 9482437

[71] Gao HN Lu HZ Cao B Du B Shang H Gan JH Clinical findings in 111 cases of influenza A (H7N9) virus infection. N Engl J Med (2013) 368:2277–85. 10.1056/NEJMoa1305584 23697469

[72] Gao R Cao B Hu Y Feng Z Wang D Hu W Human infection with a novel avian-origin influenza A (H7N9) virus. N Engl J Med (2013) 368:1888–97. 10.1056/NEJMoa1304459 23577628

[73] Rello J Pop-Vicas A. Clinical review: primary influenza viral pneumonia. Crit Care (2009) 13:235. 10.1186/cc8183 20085663 PMC2811908

[74] Hufford MM Richardson G Zhou H Manicassamy B Garcia-Sastre A Enelow RI Influenza-infected neutrophils within the infected lungs act as antigen presenting cells for anti-viral CD8(+) T cells. PLoS One (2012) 7:e46581. 10.1371/journal.pone.0046581 23056353 PMC3466305

[75] Sprenger H Meyer RG Kaufmann A Bussfeld D Rischkowsky E Gemsa D Selective induction of monocyte and not neutrophil-attracting chemokines after influenza A virus infection. J Exp Med (1996) 184:1191–6. 10.1084/jem.184.3.1191 9064338 PMC2192790

[76] Gu J Xie Z Gao Z Liu J Korteweg C Ye J H5N1 infection of the respiratory tract and beyond: a molecular pathology study. Lancet (2007) 370:1137–45. 10.1016/S0140-6736(07)61515-3 17905166 PMC7159293

[77] Chandrasekaran A Srinivasan A Raman R Viswanathan K Raguram S Tumpey TM Glycan topology determines human adaptation of avian H5N1 virus hemagglutinin. Nat Biotechnol (2008) 26:107–13. 10.1038/nbt1375 18176555

[78] Imai M Watanabe T Hatta M Das SC Ozawa M Shinya K Experimental adaptation of an influenza H5 HA confers respiratory droplet transmission to a reassortant H5 HA/H1N1 virus in ferrets. Nature (2012) 486:420–8. 10.1038/nature10831 22722205 PMC3388103

[79] de Graaf M Fouchier RA. Role of receptor binding specificity in influenza A virus transmission and pathogenesis. EMBO J (2014) 33:823–41. 10.1002/embj.201387442 24668228 PMC4194109

[80] Herfst S Schrauwen EJ Linster M Chutinimitkul S de Wit E Munster VJ Airborne transmission of influenza A/H5N1 virus between ferrets. Science (2012) 336:1534–41. 10.1126/science.1213362 22723413 PMC4810786

[81] Chan PK. Outbreak of avian influenza A(H5N1) virus infection in Hong Kong in 1997. Clin Infect Dis (2002) 34(Suppl 2):S58–64. 10.1086/338820 11938498

[82] Claas EC Osterhaus AD van Beek R De Jong JC Rimmelzwaan GF Senne DA Human influenza A H5N1 virus related to a highly pathogenic avian influenza virus. Lancet (1998) 351:472–7. 10.1016/S0140-6736(97)11212-0 9482438

[83] Korteweg C Gu J. Pathology, molecular biology, and pathogenesis of avian influenza A (H5N1) infection in humans. Am J Pathol (2008) 172:1155–70. 10.2353/ajpath.2008.070791 18403604 PMC2329826

[84] Nicholls JM Chan MC Chan WY Wong HK Cheung CY Kwong DL Tropism of avian influenza A (H5N1) in the upper and lower respiratory tract. Nat Med (2007) 13:147–9. 10.1038/nm1529 17206149

[85] Hilleman MR. Realities and enigmas of human viral influenza: pathogenesis, epidemiology and control. Vaccine (2002) 20:3068–87. 10.1016/S0264-410X(02)00254-2 12163258

[86] Carrat F Vergu E Ferguson NM Lemaitre M Cauchemez S Leach S Time lines of infection and disease in human influenza: a review of volunteer challenge studies. Am J Epidemiol (2008) 167:775–85. 10.1093/aje/kwm375 18230677

[87] Hayden FG Fritz R Lobo MC Alvord W Strober W Straus SE. Local and systemic cytokine responses during experimental human influenza A virus infection. Relation to symptom formation and host defense. J Clin Invest (1998) 101:643–9. 10.1172/JCI1355 9449698 PMC508608

[88] Skoner DP Gentile DA Patel A Doyle WJ. Evidence for cytokine mediation of disease expression in adults experimentally infected with influenza A virus. J Infect Dis (1999) 180:10–4. 10.1086/314823 10353855

[89] Kaiser L Fritz RS Straus SE Gubareva L Hayden FG. Symptom pathogenesis during acute influenza: interleukin-6 and other cytokine responses. J Med Virol (2001) 64:262–8. 10.1002/jmv.1045 11424113

[90] Francis T Stuart-Harris CH. Studies on the nasal histology of epidemic influenza virus infection in the ferret: I. The development and repair of the nasal lesion. J Exp Med (1938) 68:789–802. 10.1084/jem.68.6.789 19870817 PMC2133710

[91] Haff RF Schriver PW Stewart RC Pathogenesis of influenza in ferrets: nasal manifestations of disease. Br J Exp Pathol (1966) 47:435–44. 5924078 PMC2093728

[92] Walsh JJ Dietlein LF Low FN Burch GE Mogabgab WJ Bronchotracheal response in human influenza. Type A, Asian strain, as studied by light and electron microscopic examination of bronchoscopic biopsies. Arch Intern Med (1961) 108:376–88. 10.1001/archinte.1961.03620090048006 13782910

[93] Lee N Wong CK Chan PK Chan MC Wong RY Lun SW Cytokine response patterns in severe pandemic 2009 H1N1 and seasonal influenza among hospitalized adults. PLoS One (2011) 6:e26050. 10.1371/journal.pone.0026050 22022504 PMC3192778

[94] Glinsky GV. Genomic analysis of pandemic (H1N1) 2009 reveals association of increasing disease severity with emergence of novel hemagglutinin mutations. Cell Cycle (2010) 9:958–70. 10.4161/cc.9.5.10913 20160492

[95] Baillie GJ Galiano M Agapow PM Myers R Chiam R Gall A Evolutionary dynamics of local pandemic H1N1/2009 influenza virus lineages revealed by whole-genome analysis. J Virol (2012) 86:11–8. 10.1128/JVI.05347-11 22013031 PMC3255882

[96] Zarychanski R Stuart TL Kumar A Doucette S Elliott L Kettner J Correlates of severe disease in patients with 2009 pandemic influenza (H1N1) virus infection. CMAJ (2010) 182:257–64. 10.1503/cmaj.091884 20093297 PMC2826467

[97] Louie JK Acosta M Winter K Jean C Gavali S Schechter R Factors associated with death or hospitalization due to pandemic 2009 influenza A(H1N1) infection in California. JAMA (2009) 302:1896–902. 10.1001/jama.2009.1583 19887665

[98] Nguyen-Van-Tam JS Openshaw PJ Hashim A Gadd EM Lim WS Semple MG Risk factors for hospitalisation and poor outcome with pandemic A/H1N1 influenza: United Kingdom first wave (May-September 2009). Thorax (2010) 65:645–51. 10.1136/thx.2010.135210 20627925 PMC2921287

[99] Rosen DG Lopez AE Anzalone ML Wolf DA Derrick SM Florez LF Postmortem findings in eight cases of influenza A/H1N1. Mod Pathol (2010) 23:1449–57. 10.1038/modpathol.2010.148 20802471

[100] Hagau N Slavcovici A Gonganau DN Oltean S Dirzu DS Brezoszki ES Clinical aspects and cytokine response in severe H1N1 influenza A virus infection. Crit Care (2010) 14:R203. 10.1186/cc9324 21062445 PMC3220006

[101] Kim YH Kim JE Hyun MC. Cytokine response in pediatric patients with pandemic influenza H1N1 2009 virus infection and pneumonia: comparison with pediatric pneumonia without H1N1 2009 infection. Pediatr Pulmonol (2011) 46:1233–9. 10.1002/ppul.21496 21626718 PMC7167952

[102] Takano T Tajiri H Kashiwagi Y Kimura S Kawashima H. Cytokine and chemokine response in children with the 2009 pandemic influenza A (H1N1) virus infection. Eur J Clin Microbiol Infect Dis (2011) 30:117–20. 10.1007/s10096-010-1041-9 20820834 PMC2998638

[103] To KK Hung IF Li IW Lee KL Koo CK Yan WW Delayed clearance of viral load and marked cytokine activation in severe cases of pandemic H1N1 2009 influenza virus infection. Clin Infect Dis (2010) 50:850–9. 10.1086/650581 20136415 PMC7107930

[104] Stevens J Blixt O Glaser L Taubenberger JK Palese P Paulson JC Glycan microarray analysis of the hemagglutinins from modern and pandemic influenza viruses reveals different receptor specificities. J Mol Biol (2006) 355:1143–55. 10.1016/j.jmb.2005.11.002 16343533

[105] Morens DM Taubenberger JK Fauci AS. Predominant role of bacterial pneumonia as a cause of death in pandemic influenza: implications for pandemic influenza preparedness. J Infect Dis (2008) 198:962–70. 10.1086/591708 18710327 PMC2599911

[106] Memoli MJ Tumpey TM Jagger BW Dugan VG Sheng ZM Qi L An early ’classical’ swine H1N1 influenza virus shows similar pathogenicity to the 1918 pandemic virus in ferrets and mice. Virology (2009) 393:338–45. 10.1016/j.virol.2009.08.021 19733889 PMC2763968

[107] Kobasa D Jones SM Shinya K Kash JC Copps J Ebihara H Aberrant innate immune response in lethal infection of macaques with the 1918 influenza virus. Nature (2007) 445:319–23. 10.1038/nature05495 17230189

[108] Tumpey TM Basler CF Aguilar PV Zeng H Solorzano A Swayne DE Characterization of the reconstructed 1918 Spanish influenza pandemic virus. Science (2005) 310:77–80. 10.1126/science.1119392 16210530

[109] Wang SM Liao YT Hu YS Ho TS Shen CF Wang JR Immunophenotype expressions and cytokine profiles of influenza A H1N1 virus infection in pediatric patients in 2009. Dis Markers (2014) 2014:195453. 10.1155/2014/195453 24696530 PMC3948652

[110] Palese P. Influenza: old and new threats. Nat Med (2004) 10:S82–7. 10.1038/nm1141 15577936

[111] van den Brand JM Stittelaar KJ van Amerongen G Reperant L de Waal L Osterhaus AD Comparison of temporal and spatial dynamics of seasonal H3N2, pandemic H1N1 and highly pathogenic avian influenza H5N1 virus infections in ferrets. PLoS One (2012) 7:e42343. 10.1371/journal.pone.0042343 22905124 PMC3414522

[112] van den Brand JM Stittelaar KJ van Amerongen G Rimmelzwaan GF Simon J de Wit E Severity of pneumonia due to new H1N1 influenza virus in ferrets is intermediate between that due to seasonal H1N1 virus and highly pathogenic avian influenza H5N1 virus. J Infect Dis (2010) 201:993–9. 10.1086/651132 20187747 PMC7110095

[113] Belser JA Jayaraman A Raman R Pappas C Zeng H Cox NJ Effect of D222G mutation in the hemagglutinin protein on receptor binding, pathogenesis and transmissibility of the 2009 pandemic H1N1 influenza virus. PLoS One (2011) 6:e25091. 10.1371/journal.pone.0025091 21966421 PMC3178596

[114] Maines TR Belser JA Gustin KM van Hoeven N Zeng H Svitek N Local innate immune responses and influenza virus transmission and virulence in ferrets. J Infect Dis (2012) 205:474–85. 10.1093/infdis/jir768 22158704

[115] Maines TR Jayaraman A Belser JA Wadford DA Pappas C Zeng H Transmission and pathogenesis of swine-origin 2009 A(H1N1) influenza viruses in ferrets and mice. Science (2009) 325:484–7. 10.1126/science.1177238 19574347 PMC2953552

[116] Neumann G Ozawa M Kawaoka Y. Reverse genetics of influenza viruses. Methods Mol Biol (2012) 865:193–206. 10.1007/978-1-61779-621-0_12 22528161

[117] Abed Y Pizzorno A Hamelin ME Leung A Joubert P Couture C The 2009 pandemic H1N1 D222G hemagglutinin mutation alters receptor specificity and increases virulence in mice but not in ferrets. J Infect Dis (2011) 204:1008–16. 10.1093/infdis/jir483 21881115

[118] Camp JV Chu YK Chung DH McAllister RC Adcock RS Gerlach RL Phenotypic differences in virulence and immune response in closely related clinical isolates of influenza A 2009 H1N1 pandemic viruses in mice. PLoS One (2013) 8:e56602. 10.1371/journal.pone.0056602 23441208 PMC3575477

[119] Chutinimitkul S Herfst S Steel J Lowen AC Ye J van Riel D Virulence-associated substitution D222G in the hemagglutinin of 2009 pandemic influenza A(H1N1) virus affects receptor binding. J Virol (2010) 84:11802–13. 10.1128/JVI.01136-10 20844044 PMC2977876

[120] Itoh Y Shinya K Kiso M Watanabe T Sakoda Y Hatta M In vitro and in vivo characterization of new swine-origin H1N1 influenza viruses. Nature (2009) 460:1021–5. 10.1038/nature08260 19672242 PMC2748827

[121] Jhung MA Swerdlow D Olsen SJ Jernigan D Biggerstaff M Kamimoto L Epidemiology of 2009 pandemic influenza A (H1N1) in the United States. Clin Infect Dis (2011) 52(Suppl 1):S13–26. 10.1093/cid/ciq008 21342884

[122] Melidou A Gioula G Exindari M Chatzidimitriou D Diza E Malisiovas N. Molecular and phylogenetic analysis of the haemagglutinin gene of pandemic influenza H1N1 2009 viruses associated with severe and fatal infections. Virus Res (2010) 151:192–9. 10.1016/j.virusres.2010.05.005 20493216

[123] Meunier I Embury-Hyatt C Stebner S Gray M Bastien N Li Y Virulence differences of closely related pandemic 2009 H1N1 isolates correlate with increased inflammatory responses in ferrets. Virology (2012) 422:125–31. 10.1016/j.virol.2011.10.018 22074911

[124] Otte A Gabriel G. 2009 pandemic H1N1 influenza A virus strains display differential pathogenicity in C57BL/6J but not BALB/c mice. Virulence (2011) 2:563–6. 10.4161/viru.2.6.18148 22030859

[125] Safronetz D Rockx B Feldmann F Belisle SE Palermo RE Brining D Pandemic swine-origin H1N1 influenza A virus isolates show heterogeneous virulence in macaques. J Virol (2011) 85:1214–23. 10.1128/JVI.01848-10 21084481 PMC3020514

[126] Ye J Sorrell EM Cai Y Shao H Xu K Pena L Variations in the hemagglutinin of the 2009 H1N1 pandemic virus: potential for strains with altered virulence phenotype? PLoS Pathog (2010) 6:e1001145. 10.1371/journal.ppat.1001145 20976194 PMC2954835

[127] Gubareva LV McCullers JA Bethell RC Webster RG. Characterization of influenza A/HongKong/156/97 (H5N1) virus in a mouse model and protective effect of zanamivir on H5N1 infection in mice. J Infect Dis (1998) 178:1592–6. 10.1086/314515 9815209

[128] Shortridge KF Zhou NN Guan Y Gao P Ito T Kawaoka Y Characterization of avian H5N1 influenza viruses from poultry in Hong Kong. Virology (1998) 252:331–42. 10.1006/viro.1998.9488 9878612

[129] Chen Y Liang W Yang S Wu N Gao H Sheng J Human infections with the emerging avian influenza A H7N9 virus from wet market poultry: clinical analysis and characterisation of viral genome. Lancet (2013) 381:1916–25. 10.1016/S0140-6736(13)60903-4 23623390 PMC7134567

[130] Wong SS Yuen KY. Avian influenza virus infections in humans. Chest (2006) 129:156–68. 10.1378/chest.129.1.156 16424427 PMC7094746

[131] Fouchier RA Schneeberger PM Rozendaal FW Broekman JM Kemink SA Munster V Avian influenza A virus (H7N7) associated with human conjunctivitis and a fatal case of acute respiratory distress syndrome. Proc Natl Acad Sci U S A (2004) 101:1356–61. 10.1073/pnas.0308352100 14745020 PMC337057

[132] Chen E Chen Y Fu L Chen Z Gong Z Mao H Human infection with avian influenza A(H7N9) virus re-emerges in China in winter 2013. Euro Surveill (2013) 18:20616. 10.2807/1560-7917.ES2013.18.43.20616 24176616

[133] Shinya K Gao Y Cilloniz C Suzuki Y Fujie M Deng G Integrated clinical, pathologic, virologic, and transcriptomic analysis of H5N1 influenza virus-induced viral pneumonia in the rhesus macaque. J Virol (2012) 86:6055–66. 10.1128/JVI.00365-12 22491448 PMC3372212

[134] Zhou J Wang D Gao R Zhao B Song J Qi X Biological features of novel avian influenza A (H7N9) virus. Nature (2013) 499:500–3. 10.1038/nature12379 23823727

[135] Bouvier NM Lowen AC. Animal models for influenza virus pathogenesis and transmission. Viruses (2010) 2:1530–63. 10.3390/v20801530 21442033 PMC3063653

[136] Long JP Kotur MS Stark GV Warren RL Kasoji M Craft JL Accumulation of CD11b(+)Gr-1(+) cells in the lung, blood and bone marrow of mice infected with highly pathogenic H5N1 and H1N1 influenza viruses. Arch Virol (2013) 158:1305–22. 10.1007/s00705-012-1593-3 23397329 PMC5533288

[137] Zhu H Wang D Kelvin DJ Li L Zheng Z Yoon SW Infectivity, transmission, and pathology of human-isolated H7N9 influenza virus in ferrets and pigs. Science (2013) 341:183–6. 10.1126/science.1239844 23704376

[138] Tate MD Ioannidis LJ Croker B Brown LE Brooks AG Reading PC. The role of neutrophils during mild and severe influenza virus infections of mice. PLoS One (2011) 6:e17618. 10.1371/journal.pone.0017618 21423798 PMC3056712

[139] Narasaraju T Yang E Samy RP Ng HH Poh WP Liew AA Excessive neutrophils and neutrophil extracellular traps contribute to acute lung injury of influenza pneumonitis. Am J Pathol (2011) 179:199–210. 10.1016/j.ajpath.2011.03.013 21703402 PMC3123873

[140] Tate MD Brooks AG Reading PC. The role of neutrophils in the upper and lower respiratory tract during influenza virus infection of mice. Respir Res (2008) 9:57. 10.1186/1465-9921-9-57 18671884 PMC2526083

[141] Bradley LM Douglass MF Chatterjee D Akira S Baaten BJ. Matrix metalloprotease 9 mediates neutrophil migration into the airways in response to influenza virus-induced toll-like receptor signaling. PLoS Pathog (2012) 8:e1002641. 10.1371/journal.ppat.1002641 22496659 PMC3320598

[142] Seo SU Kwon HJ Song JH Byun YH Seong BL Kawai T MyD88 signaling is indispensable for primary influenza A virus infection but dispensable for secondary infection. J Virol (2010) 84:12713–22. 10.1128/JVI.01675-10 20943980 PMC3004294

[143] Walsh KB Teijaro JR Brock LG Fremgen DM Collins PL Rosen H Animal model of respiratory syncytial virus: CD8+ T cells cause a cytokine storm that is chemically tractable by sphingosine-1-phosphate 1 receptor agonist therapy. J Virol (2014) 88:6281–93. 10.1128/JVI.00464-14 24672024 PMC4093868

[144] Oldstone MB Rosen H. Cytokine storm plays a direct role in the morbidity and mortality from influenza virus infection and is chemically treatable with a single sphingosine-1-phosphate agonist molecule. Curr Top Microbiol Immunol (2014) 378:129–47. 10.1007/978-3-319-05879-5_6 24728596 PMC7121493

[145] Borregaard N Sorensen OE Theilgaard-Monch K. Neutrophil granules: a library of innate immunity proteins. Trends Immunol (2007) 28:340–5. 10.1016/j.it.2007.06.002 17627888

[146] Brinkmann V Reichard U Goosmann C Fauler B Uhlemann Y Weiss DS Neutrophil extracellular traps kill bacteria. Science (2004) 303:1532–5. 10.1126/science.1092385 15001782

[147] Prince LR Whyte MK Sabroe I Parker LC. The role of TLRs in neutrophil activation. Curr Opin Pharmacol (2011) 11:397–403. 10.1016/j.coph.2011.06.007 21741310

[148] Thomas CJ Schroder K. Pattern recognition receptor function in neutrophils. Trends Immunol (2013) 34:317–28. 10.1016/j.it.2013.02.008 23540649

[149] Bjornson AB Mellencamp MA Schiff GM. Complement is activated in the upper respiratory tract during influenza virus infection. Am Rev Respir Dis (1991) 143:1062–6. 10.1164/ajrccm/143.5_Pt_1.1062 2024815

[150] Fritz RS Hayden FG Calfee DP Cass LM Peng AW Alvord WG Nasal cytokine and chemokine responses in experimental influenza A virus infection: results of a placebo-controlled trial of intravenous zanamivir treatment. J Infect Dis (1999) 180:586–93. 10.1086/314938 10438343

[151] Lee NL. Role of cytokines and chemokines in severe and complicated influenza infections. Hong Kong Med J (2009) 15(Suppl 8):38–41. 20393212

[152] Lee N Chan PK Wong CK Wong KT Choi KW Joynt GM Viral clearance and inflammatory response patterns in adults hospitalized for pandemic 2009 influenza A(H1N1) virus pneumonia. Antivir Ther (2011) 16:237–47. 10.3851/IMP1722 21447873

[153] Garcia CC Weston-Davies W Russo RC Tavares LP Rachid MA Alves-Filho JC Complement C5 activation during influenza A infection in mice contributes to neutrophil recruitment and lung injury. PLoS One (2013) 8:e64443. 10.1371/journal.pone.0064443 23696894 PMC3655967

[154] Schmitz N Kurrer M Bachmann MF Kopf M. Interleukin-1 is responsible for acute lung immunopathology but increases survival of respiratory influenza virus infection. J Virol (2005) 79:6441–8. 10.1128/JVI.79.10.6441-6448.2005 15858027 PMC1091664

[155] Ichinohe T Lee HK Ogura Y Flavell R Iwasaki A. Inflammasome recognition of influenza virus is essential for adaptive immune responses. J Exp Med (2009) 206:79–87. 10.1084/jem.20081667 19139171 PMC2626661

[156] Crowe CR Chen K Pociask DA Alcorn JF Krivich C Enelow RI Critical role of IL-17RA in immunopathology of influenza infection. J Immunol (2009) 183:5301–10. 10.4049/jimmunol.0900995 19783685 PMC3638739

[157] Ichikawa A Kuba K Morita M Chida S Tezuka H Hara H CXCL10-CXCR3 enhances the development of neutrophil-mediated fulminant lung injury of viral and nonviral origin. Am J Respir Crit Care Med (2013) 187:65–77. 10.1164/rccm.201203-0508OC 23144331 PMC3927876

[158] Schliehe C Flynn EK Vilagos B Richson U Swaminathan S Bosnjak B The methyltransferase Setdb2 mediates virus-induced susceptibility to bacterial superinfection. Nat Immunol (2015) 16:67–74. 10.1038/ni.3046 25419628 PMC4320687

[159] Wheeler JL Martin KC Lawrence BP. Novel cellular targets of AhR underlie alterations in neutrophilic inflammation and inducible nitric oxide synthase expression during influenza virus infection. J Immunol (2013) 190:659–68. 10.4049/jimmunol.1201341 23233726 PMC3538960

[160] Teske S Bohn AA Hogaboam JP Lawrence BP. Aryl hydrocarbon receptor targets pathways extrinsic to bone marrow cells to enhance neutrophil recruitment during influenza virus infection. Toxicol Sci (2008) 102:89–99. 10.1093/toxsci/kfm282 18007012 PMC2919339

[161] Neff-LaFord H Teske S Bushnell TP Lawrence BP. Aryl hydrocarbon receptor activation during influenza virus infection unveils a novel pathway of IFN-gamma production by phagocytic cells. J Immunol (2007) 179:247–55. 10.4049/jimmunol.179.1.247 17579044

[162] Teske S Bohn AA Regal JF Neumiller JJ Lawrence BP. Activation of the aryl hydrocarbon receptor increases pulmonary neutrophilia and diminishes host resistance to influenza A virus. Am J Physiol Lung Cell Mol Physiol (2005) 289:L111–24. 10.1152/ajplung.00318.2004 15792965

[163] Ratcliffe D Migliorisi G Cramer E. Translocation of influenza virus by migrating neutrophils. Cell Mol Biol (1992) 38:63–70. 1559245

[164] Hayashi F Means TK Luster AD. Toll-like receptors stimulate human neutrophil function. Blood (2003) 102:2660–9. 10.1182/blood-2003-04-1078 12829592

[165] Hattermann K Picard S Borgeat M Leclerc P Pouliot M Borgeat P. The toll-like receptor 7/8-ligand resiquimod (R-848) primes human neutrophils for leukotriene B4, prostaglandin E2 and platelet-activating factor biosynthesis. FASEB J (2007) 21:1575–85. 10.1096/fj.06-7457com 17264163

[166] Tamassia N Le Moigne V Rossato M Donini M McCartney S Calzetti F Activation of an immunoregulatory and antiviral gene expression program in poly(I:C)-transfected human neutrophils. J Immunol (2008) 181:6563–73. 10.4049/jimmunol.181.9.6563 18941247

[167] Le Goffic R Balloy V Lagranderie M Alexopoulou L Escriou N Flavell R Detrimental contribution of the toll-like receptor (TLR)3 to influenza A virus-induced acute pneumonia. PLoS Pathog (2006) 2:e53. 10.1371/journal.ppat.0020053 16789835 PMC1475659

[168] Leung YH Nicholls JM Ho CK Sia SF Mok CK Valkenburg SA Highly pathogenic avian influenza A H5N1 and pandemic H1N1 virus infections have different phenotypes in toll-like receptor 3 knockout mice. J Gen Virol (2014) 95:1870–9. 10.1099/vir.0.066258-0 24878639 PMC4135086

[169] Andonegui G Goyert SM Kubes P. Lipopolysaccharide-induced leukocyte-endothelial cell interactions: a role for CD14 versus toll-like receptor 4 within microvessels. J Immunol (2002) 169:2111–9. 10.4049/jimmunol.169.4.2111 12165539

[170] Kolb JP Casella CR SenGupta S Chilton PM Mitchell TC. Type I interferon signaling contributes to the bias that toll-like receptor 4 exhibits for signaling mediated by the adaptor protein TRIF. Sci Signal (2014) 7:ra108. 10.1126/scisignal.2005442 25389373 PMC4459894

[171] van Bruggen R Drewniak A Tool AT Jansen M van Houdt M Geissler J Toll-like receptor responses in IRAK-4-deficient neutrophils. J Innate Immun (2010) 2:280–7. 10.1159/000268288 20375545

[172] Hartshorn K Chang D Rust K White M Heuser J Crouch E. Interactions of recombinant human pulmonary surfactant protein D and SP-D multimers with influenza A. Am J Physiol (1996) 271:L753–62. 8944718 10.1152/ajplung.1996.271.5.L753

[173] Hartshorn KL Crouch EC White MR Eggleton P Tauber AI Chang D Evidence for a protective role of pulmonary surfactant protein D (SP-D) against influenza A viruses. J Clin Invest (1994) 94:311–9. 10.1172/JCI117323 8040272 PMC296311

[174] Hartshorn KL White MR Voelker DR Coburn J Zaner K Crouch EC. Mechanism of binding of surfactant protein D to influenza A viruses: importance of binding to haemagglutinin to antiviral activity. Biochem J (2000) 351(Pt 2):449–58. 10.1042/bj3510449 11023831 PMC1221381

[175] LeVine AM Whitsett JA Hartshorn KL Crouch EC Korfhagen TR. Surfactant protein D enhances clearance of influenza A virus from the lung in vivo. J Immunol (2001) 167:5868–73. 10.4049/jimmunol.167.10.5868 11698462

[176] Qi L Kash JC Dugan VG Jagger BW Lau YF Sheng ZM The ability of pandemic influenza virus hemagglutinins to induce lower respiratory pathology is associated with decreased surfactant protein D binding. Virology (2011) 412:426–34. 10.1016/j.virol.2011.01.029 21334038 PMC3060949

[177] van Eijk M Bruinsma L Hartshorn KL White MR Rynkiewicz MJ Seaton BA Introduction of N-linked glycans in the lectin domain of surfactant protein D: impact on interactions with influenza A viruses. J Biol Chem (2011) 286:20137–51. 10.1074/jbc.M111.224469 21489996 PMC3121484

[178] Hartshorn KL White MR Tecle T Holmskov U Crouch EC. Innate defense against influenza A virus: activity of human neutrophil defensins and interactions of defensins with surfactant protein D. J Immunol (2006) 176:6962–72. 10.4049/jimmunol.176.11.6962 16709857

[179] Tecle T White MR Gantz D Crouch EC Hartshorn KL. Human neutrophil defensins increase neutrophil uptake of influenza A virus and bacteria and modify virus-induced respiratory burst responses. J Immunol (2007) 178:8046–52. 10.4049/jimmunol.178.12.8046 17548642

[180] Doss M White MR Tecle T Gantz D Crouch EC Jung G Interactions of alpha-, beta-, and theta-defensins with influenza A virus and surfactant protein D. J Immunol (2009) 182:7878–87. 10.4049/jimmunol.0804049 19494312

[181] Salvatore M Garcia-Sastre A Ruchala P Lehrer RI Chang T Klotman ME. alpha-Defensin inhibits influenza virus replication by cell-mediated mechanism(s). J Infect Dis (2007) 196:835–43. 10.1086/521027 17703413

[182] Daher KA Selsted ME Lehrer RI. Direct inactivation of viruses by human granulocyte defensins. J Virol (1986) 60:1068–74. 3023659 10.1128/jvi.60.3.1068-1074.1986 PMC253347

[183] Ratcliffe DR Michl J Cramer EB. Neutrophils do not bind to or phagocytize human immune complexes formed with influenza virus. Blood (1993) 82:1639–46. 8364212

[184] Fujisawa H. Neutrophils play an essential role in cooperation with antibody in both protection against and recovery from pulmonary infection with influenza virus in mice. J Virol (2008) 82:2772–83. 10.1128/JVI.01210-07 18184718 PMC2258992

[185] Hartshorn KL Liou LS White MR Kazhdan MM Tauber JL Tauber AI. Neutrophil deactivation by influenza A virus. Role of hemagglutinin binding to specific sialic acid-bearing cellular proteins. J Immunol (1995) 154:3952–60. 7706733

[186] Ivan FX Tan KS Phoon MC Engelward BP Welsch RE Rajapakse JC Neutrophils infected with highly virulent influenza H3N2 virus exhibit augmented early cell death and rapid induction of type I interferon signaling pathways. Genomics (2013) 101:101–12. 10.1016/j.ygeno.2012.11.008 23195410

[187] Wang JP Bowen GN Padden C Cerny A Finberg RW Newburger PE Toll-like receptor-mediated activation of neutrophils by influenza A virus. Blood (2008) 112:2028–34. 10.1182/blood-2008-01-132860 18544685 PMC2518904

[188] Allen IC Moore CB Schneider M Lei Y Davis BK Scull MA NLRX1 protein attenuates inflammatory responses to infection by interfering with the RIG-I-MAVS and TRAF6-NF-kappaB signaling pathways. Immunity (2011) 34:854–65. 10.1016/j.immuni.2011.03.026 21703540 PMC3166771

[189] Jaworska J Coulombe F Downey J Tzelepis F Shalaby K Tattoli I NLRX1 prevents mitochondrial induced apoptosis and enhances macrophage antiviral immunity by interacting with influenza virus PB1-F2 protein. Proc Natl Acad Sci U S A (2014) 111:E2110–9. 10.1073/pnas.1322118111 24799673 PMC4034189

[190] Stasakova J Ferko B Kittel C Sereinig S Romanova J Katinger H Influenza A mutant viruses with altered NS1 protein function provoke caspase-1 activation in primary human macrophages, resulting in fast apoptosis and release of high levels of interleukins 1beta and 18. J Gen Virol (2005) 86:185–95. 10.1099/vir.0.80422-0 15604446

[191] Allen IC Scull MA Moore CB Holl EK McElvania-TeKippe E Taxman DJ The NLRP3 inflammasome mediates in vivo innate immunity to influenza A virus through recognition of viral RNA. Immunity (2009) 30:556–65. 10.1016/j.immuni.2009.02.005 19362020 PMC2803103

[192] Belisle SE Tisoncik JR Korth MJ Carter VS Proll SC Swayne DE Genomic profiling of tumor necrosis factor alpha (TNF-alpha) receptor and interleukin-1 receptor knockout mice reveals a link between TNF-alpha signaling and increased severity of 1918 pandemic influenza virus infection. J Virol (2010) 84:12576–88. 10.1128/JVI.01310-10 20926563 PMC3004331

[193] Huang CH Chen CJ Yen CT Yu CP Huang PN Kuo RL Caspase-1 deficient mice are more susceptible to influenza A virus infection with PA variation. J Infect Dis (2013) 208:1898–905. 10.1093/infdis/jit381 23901080

[194] Perrone LA Szretter KJ Katz JM Mizgerd JP Tumpey TM. Mice lacking both TNF and IL-1 receptors exhibit reduced lung inflammation and delay in onset of death following infection with a highly virulent H5N1 virus. J Infect Dis (2010) 202:1161–70. 10.1086/656365 20815704 PMC2941567

[195] Szretter KJ Gangappa S Lu X Smith C Shieh WJ Zaki SR Role of host cytokine responses in the pathogenesis of avian H5N1 influenza viruses in mice. J Virol (2007) 81:2736–44. 10.1128/JVI.02336-06 17182684 PMC1866007

[196] Abramson JS Lewis JC Lyles DS Heller KA Mills EL Bass DA. Inhibition of neutrophil lysosome-phagosome fusion associated with influenza virus infection in vitro. Role in depressed bactericidal activity. J Clin Invest (1982) 69:1393–7. 10.1172/JCI110580 7085879 PMC370213

[197] Walzog B Schuppan D Heimpel C Hafezi-Moghadam A Gaehtgens P Ley K. The leukocyte integrin Mac-1 (CD11b/CD18) contributes to binding of human granulocytes to collagen. Exp Cell Res (1995) 218:28–38. 10.1006/excr.1995.1127 7737365

[198] Nathan C. Neutrophils and immunity: challenges and opportunities. Nat Rev Immunol (2006) 6:173–82. 10.1038/nri1785 16498448

[199] Sugamata R Dobashi H Nagao T Yamamoto K Nakajima N Sato Y Contribution of neutrophil-derived myeloperoxidase in the early phase of fulminant acute respiratory distress syndrome induced by influenza virus infection. Microbiol Immunol (2012) 56:171–82. 10.1111/j.1348-0421.2011.00424.x 22211924

[200] Yamamoto K Miyoshi-Koshio T Utsuki Y Mizuno S Suzuki K. Virucidal activity and viral protein modification by myeloperoxidase: a candidate for defense factor of human polymorphonuclear leukocytes against influenza virus infection. J Infect Dis (1991) 164:8–14. 10.1093/infdis/164.1.8 1647427

[201] Foong RE Sly PD Larcombe AN Zosky GR. No role for neutrophil elastase in influenza-induced cellular recruitment, cytokine production or airway hyperresponsiveness in mice. Respir Physiol Neurobiol (2010) 173:164–70. 10.1016/j.resp.2010.08.003 20696285

[202] Abramson JS Mills EL Giebink GS Quie PG. Depression of monocyte and polymorphonuclear leukocyte oxidative metabolism and bactericidal capacity by influenza A virus. Infect Immun (1982) 35:350–5. 7054126 10.1128/iai.35.1.350-355.1982 PMC351036

[203] Schwarz KB. Oxidative stress during viral infection: a review. Free Radic Biol Med (1996) 21:641–9. 10.1016/0891-5849(96)00131-1 8891667

[204] Knobil K Choi AM Weigand GW Jacoby DB. Role of oxidants in influenza virus-induced gene expression. Am J Physiol (1998) 274:L134–42. 9458811 10.1152/ajplung.1998.274.1.L134

[205] Lazrak A Iles KE Liu G Noah DL Noah JW Matalon S. Influenza virus M2 protein inhibits epithelial sodium channels by increasing reactive oxygen species. FASEB J (2009) 23:3829–42. 10.1096/fj.09-135590 19596899 PMC2775009

[206] Snelgrove RJ Edwards L Rae AJ Hussell T. An absence of reactive oxygen species improves the resolution of lung influenza infection. Eur J Immunol (2006) 36:1364–73. 10.1002/eji.200635977 16703568

[207] Cooper JA Jr Carcelen R Culbreth R. Effects of influenza A nucleoprotein on polymorphonuclear neutrophil function. J Infect Dis (1996) 173:279–84. 10.1093/infdis/173.2.279 8568286

[208] Buffinton GD Christen S Peterhans E Stocker R. Oxidative stress in lungs of mice infected with influenza A virus. Free Radic Res Commun (1992) 16:99–110. 10.3109/10715769209049163 1321077

[209] Perrone LA Belser JA Wadford DA Katz JM Tumpey TM. Inducible nitric oxide contributes to viral pathogenesis following highly pathogenic influenza virus infection in mice. J Infect Dis (2013) 207:1576–84. 10.1093/infdis/jit062 23420903

[210] Suliman HB Ryan LK Bishop L Folz RJ. Prevention of influenza-induced lung injury in mice overexpressing extracellular superoxide dismutase. Am J Physiol Lung Cell Mol Physiol (2001) 280:L69–78. 11133496 10.1152/ajplung.2001.280.1.L69

[211] Vlahos R Stambas J Bozinovski S Broughton BR Drummond GR Selemidis S. Inhibition of Nox2 oxidase activity ameliorates influenza A virus-induced lung inflammation. PLoS Pathog (2011) 7:e1001271. 10.1371/journal.ppat.1001271 21304882 PMC3033375

[212] Zhang WJ Wei H Tien YT Frei B Genetic ablation of phagocytic NADPH oxidase in mice limits TNFalpha-induced inflammation in the lungs but not other tissues. Free Radic Biol Med (2011) 50:1517–25. 10.1016/j.freeradbiomed.2011.02.027 21376114 PMC3090478

[213] Akaike T Noguchi Y Ijiri S Setoguchi K Suga M Zheng YM Pathogenesis of influenza virus-induced pneumonia: involvement of both nitric oxide and oxygen radicals. Proc Natl Acad Sci U S A (1996) 93:2448–53. 10.1073/pnas.93.6.2448 8637894 PMC39817

[214] Imai Y Kuba K Neely GG Yaghubian-Malhami R Perkmann T van Loo G Identification of oxidative stress and toll-like receptor 4 signaling as a key pathway of acute lung injury. Cell (2008) 133:235–49. 10.1016/j.cell.2008.02.043 18423196 PMC7112336

[215] Nhu QM Shirey K Teijaro JR Farber DL Netzel-Arnett S Antalis TM Novel signaling interactions between proteinase-activated receptor 2 and toll-like receptors in vitro and in vivo. Mucosal Immunol (2010) 3:29–39. 10.1038/mi.2009.120 19865078 PMC2851245

[216] Shirey KA Lai W Scott AJ Lipsky M Mistry P Pletneva LM The TLR4 antagonist Eritoran protects mice from lethal influenza infection. Nature (2013) 497:498–502. 10.1038/nature12118 23636320 PMC3725830

[217] Fuchs TA Abed U Goosmann C Hurwitz R Schulze I Wahn V Novel cell death program leads to neutrophil extracellular traps. J Cell Biol (2007) 176:231–41. 10.1083/jcb.200606027 17210947 PMC2063942

[218] Kaplan MJ Radic M. Neutrophil extracellular traps: double-edged swords of innate immunity. J Immunol (2012) 189:2689–95. 10.4049/jimmunol.1201719 22956760 PMC3439169

[219] Urban CF Reichard U Brinkmann V Zychlinsky A. Neutrophil extracellular traps capture and kill *Candida albicans* yeast and hyphal forms. Cell Microbiol (2006) 8:668–76. 10.1111/j.1462-5822.2005.00659.x 16548892

[220] Guimaraes-Costa AB Nascimento MT Froment GS Soares RP Morgado FN Conceicao-Silva F *Leishmania amazonensis* promastigotes induce and are killed by neutrophil extracellular traps. Proc Natl Acad Sci U S A (2009) 106:6748–53. 10.1073/pnas.0900226106 19346483 PMC2672475

[221] Agraz-Cibrian JM Giraldo DM Mary FM Urcuqui-Inchima S. Understanding the molecular mechanisms of NETs and their role in antiviral innate immunity. Virus Res (2017) 228:124–33. 10.1016/j.virusres.2016.11.033 27923601

[222] Kessenbrock K Krumbholz M Schonermarck U Back W Gross WL Werb Z Netting neutrophils in autoimmune small-vessel vasculitis. Nat Med (2009) 15:623–5. 10.1038/nm.1959 19448636 PMC2760083

[223] Marcos V Zhou Z Yildirim AO Bohla A Hector A Vitkov L CXCR2 mediates NADPH oxidase-independent neutrophil extracellular trap formation in cystic fibrosis airway inflammation. Nat Med (2010) 16:1018–23. 10.1038/nm.2209 20818377

[224] Hakkim A Furnrohr BG Amann K Laube B Abed UA Brinkmann V Impairment of neutrophil extracellular trap degradation is associated with lupus nephritis. Proc Natl Acad Sci U S A (2010) 107:9813–8. 10.1073/pnas.0909927107 20439745 PMC2906830

[225] Raftery MJ Lalwani P Krautkrmer E Peters T Scharffetter-Kochanek K Kruger R beta2 integrin mediates hantavirus-induced release of neutrophil extracellular traps. J Exp Med (2014) 211:1485–97. 10.1084/jem.20131092 24889201 PMC4076588

[226] Hemmers S Teijaro JR Arandjelovic S Mowen KA. PAD4-mediated neutrophil extracellular trap formation is not required for immunity against influenza infection. PLoS One (2011) 6:e22043. 10.1371/journal.pone.0022043 21779371 PMC3133614

[227] Eruslanov EB Lyadova IV Kondratieva TK Majorov KB Scheglov IV Orlova MO Neutrophil responses to *Mycobacterium tuberculosis* infection in genetically susceptible and resistant mice. Infect Immun (2005) 73:1744–53. 10.1128/IAI.73.3.1744-1753.2005 15731075 PMC1064912

[228] Seiler P Aichele P Bandermann S Hauser AE Lu B Gerard NP Early granuloma formation after aerosol *Mycobacterium tuberculosis* infection is regulated by neutrophils via CXCR3-signaling chemokines. Eur J Immunol (2003) 33:2676–86. 10.1002/eji.200323956 14515251

[229] Kennedy JL Turner RB Braciale T Heymann PW Borish L Pathogenesis of rhinovirus infection. Curr Opin Virol (2012) 2:287–93. 10.1016/j.coviro.2012.03.008 22542099 PMC3378761

[230] Levandowski RA Weaver CW Jackson GG. Nasal-secretion leukocyte populations determined by flow cytometry during acute rhinovirus infection. J Med Virol (1988) 25:423–32. 10.1002/jmv.1890250406 2844984

[231] Turner RB The role of neutrophils in the pathogenesis of rhinovirus infections. Pediatr Infect Dis J (1990) 9:832–5. 10.1097/00006454-199011000-00011 2175875

[232] Domachowske JB Rosenberg HF. Respiratory syncytial virus infection: immune response, immunopathogenesis, and treatment. Clin Microbiol Rev (1999) 12:298–309. 10194461 10.1128/cmr.12.2.298 PMC88919

[233] Pickles RJ DeVincenzo JP. Respiratory syncytial virus (RSV) and its propensity for causing bronchiolitis. J Pathol (2015) 235:266–76. 10.1002/path.4462 25302625 PMC5638117

[234] Schildgen V van den Hoogen B Fouchier R Tripp RA Alvarez R Manoha C Human metapneumovirus: lessons learned over the first decade. Clin Microbiol Rev (2011) 24:734–54. 10.1128/CMR.00015-11 21976607 PMC3194831

[235] Vargas SO Kozakewich HP Perez-Atayde AR McAdam AJ. Pathology of human metapneumovirus infection: insights into the pathogenesis of a newly identified respiratory virus. Pediatr Dev Pathol (2004) 7:478–86; discussion 421. 10.1007/s10024-004-1011-2 15547771

[236] Hooper P Zaki S Daniels P Middleton D. Comparative pathology of the diseases caused by Hendra and Nipah viruses. Microbes Infect (2001) 3:315–22. 10.1016/S1286-4579(01)01385-5 11334749

[237] Koh YY Jung da E Koh JY Kim JY Yoo Y Kim CK. Bronchoalveolar cellularity and interleukin-8 levels in measles bronchiolitis obliterans. Chest (2007) 131:1454–60. 10.1378/chest.06-0188 17494793

[238] Downham MA Gardner PS McQuillin J Ferris JA. Role of respiratory viruses in childhood mortality. Br Med J (1975) 1:235–9. 10.1136/bmj.1.5952.235 163114 PMC1672037

[239] Zaki SR Greer PW Coffield LM Goldsmith CS Nolte KB Foucar K Hantavirus pulmonary syndrome. Pathogenesis of an emerging infectious disease. Am J Pathol (1995) 146:552–79. 7887439 PMC1869168

[240] Wu W Booth JL Duggan ES Patel KB Coggeshall KM Metcalf JP. Human lung innate immune cytokine response to adenovirus type 7. J Gen Virol (2010) 91:1155–63. 10.1099/vir.0.017905-0 20071488 PMC4091184

[241] Pyrc K Berkhout B van der Hoek L The novel human coronaviruses NL63 and HKU1. J Virol (2007) 81:3051–7. 10.1128/JVI.01466-06 17079323 PMC1866027

[242] Moolenaar RL Dalton C Lipman HB Umland ET Gallaher M Duchin JS Clinical features that differentiate hantavirus pulmonary syndrome from three other acute respiratory illnesses. Clin Infect Dis (1995) 21:643–9. 10.1093/clinids/21.3.643 8527558

[243] Smits SL van den Brand JM de Lang A Leijten LM van Ijcken WF van Amerongen G Distinct severe acute respiratory syndrome coronavirus-induced acute lung injury pathways in two different nonhuman primate species. J Virol (2011) 85:4234–45. 10.1128/JVI.02395-10 21325418 PMC3126247

[244] Schomacker H Schaap-Nutt A Collins PL Schmidt AC Pathogenesis of acute respiratory illness caused by human parainfluenza viruses. Curr Opin Virol (2012) 2:294–9. 10.1016/j.coviro.2012.02.001 22709516 PMC3514439

[245] Jiang Y Xu J Zhou C Wu Z Zhong S Liu J Characterization of cytokine/chemokine profiles of severe acute respiratory syndrome. Am J Respir Crit Care Med (2005) 171:850–7. 10.1164/rccm.200407-857OC 15657466

[246] Bataki EL Evans GS Everard ML. Respiratory syncytial virus and neutrophil activation. Clin Exp Immunol (2005) 140:470–7. 10.1111/j.1365-2249.2005.02780.x 15932508 PMC1809401

[247] Kolaczkowska E Kubes P. Neutrophil recruitment and function in health and inflammation. Nat Rev Immunol (2013) 13:159–75. 10.1038/nri3399 23435331

[248] van Kempen M Bachert C Van Cauwenberge P. An update on the pathophysiology of rhinovirus upper respiratory tract infections. Rhinology (1999) 37:97–103. 10567986

[249] Winther B. Effects on the nasal mucosa of upper respiratory viruses (common cold). Dan Med Bull (1994) 41:193–204. 8039434

[250] Winther B Gwaltney JM Jr Mygind N Hendley JO. Viral-induced rhinitis. Am J Rhinol (1998) 12:17–20. 10.2500/105065898782102954 9513654

[251] Racaniello V Picornaviridae: the viruses and their replication. 5th ed In: Knipe DM Howley PM, editors. Fields Virology. (Vol. 1), Philadelphia, PA: Lippincott Williams and Wilkins (2007). p. 795–838.

[252] Gern JE Busse WW. Association of rhinovirus infections with asthma. Clin Microbiol Rev (1999) 12:9–18. 9880472 10.1128/cmr.12.1.9 PMC88904

[253] Gern JE Martin MS Anklam KA Shen K Roberg KA Carlson-Dakes KT Relationships among specific viral pathogens, virus-induced interleukin-8, and respiratory symptoms in infancy. Pediatr Allergy Immunol (2002) 13:386–93. 10.1034/j.1399-3038.2002.01093.x 12485313

[254] Murawski MR Bowen GN Cerny AM Anderson LJ Haynes LM Tripp RA Respiratory syncytial virus activates innate immunity through toll-like receptor 2. J Virol (2009) 83:1492–500. 10.1128/JVI.00671-08 19019963 PMC2620898

[255] Loo YM Fornek J Crochet N Bajwa G Perwitasari O Martinez-Sobrido L Distinct RIG-I and MDA5 signaling by RNA viruses in innate immunity. J Virol (2008) 82:335–45. 10.1128/JVI.01080-07 17942531 PMC2224404

[256] Mibayashi M Martinez-Sobrido L Loo YM Cardenas WB Gale M Jr Garcia-Sastre A. Inhibition of retinoic acid-inducible gene I-mediated induction of beta interferon by the NS1 protein of influenza A virus. J Virol (2007) 81:514–24. 10.1128/JVI.01265-06 17079289 PMC1797471

[257] Kato H Sato S Yoneyama M Yamamoto M Uematsu S Matsui K Cell type-specific involvement of RIG-I in antiviral response. Immunity (2005) 23:19–28. 10.1016/j.immuni.2005.04.010 16039576

[258] Koyama S Ishii KJ Kumar H Tanimoto T Coban C Uematsu S Differential role of TLR- and RLR-signaling in the immune responses to influenza A virus infection and vaccination. J Immunol (2007) 179:4711–20. 10.4049/jimmunol.179.7.4711 17878370

[259] Kelly JT Busse WW. Host immune responses to rhinovirus: mechanisms in asthma. J Allergy Clin Immunol (2008) 122:671–82; quiz 683–74. 10.1016/j.jaci.2008.08.013 19014757 PMC3927944

[260] Honda K Taniguchi T. IRFs: master regulators of signalling by toll-like receptors and cytosolic pattern-recognition receptors. Nat Rev Immunol (2006) 6:644–58. 10.1038/nri1900 16932750

[261] Dixit E Kagan JC. Intracellular pathogen detection by RIG-I-like receptors. Adv Immunol (2013) 117:99–125. 10.1016/B978-0-12-410524-9.00004-9 23611287 PMC3947775

[262] Zinman G Brower-Sinning R Emeche CH Ernst J Huang GT Mahony S Large scale comparison of innate responses to viral and bacterial pathogens in mouse and macaque. PLoS One (2011) 6:e22401. 10.1371/journal.pone.0022401 21789257 PMC3138787

[263] Diebold SS Kaisho T Hemmi H Akira S Reis e Sousa C. Innate antiviral responses by means of TLR7-mediated recognition of single-stranded RNA. Science (2004) 303:1529–31. 10.1126/science.1093616 14976261

[264] Byeon JH Lee JC Choi IS Yoo Y Park SH Choung JT. Comparison of cytokine responses in nasopharyngeal aspirates from children with viral lower respiratory tract infections. Acta Paediatr (2009) 98:725–30. 10.1111/j.1651-2227.2008.01208.x 19183120 PMC7159639

[265] Sung RY Hui SH Wong CK Lam CW Yin J. A comparison of cytokine responses in respiratory syncytial virus and influenza A infections in infants. Eur J Pediatr (2001) 160:117–22. 10.1007/s004310000676 11271383

[266] Shirey KA Pletneva LM Puche AC Keegan AD Prince GA Blanco JC Control of RSV-induced lung injury by alternatively activated macrophages is IL-4R alpha-, TLR4-, and IFN-beta-dependent. Mucosal Immunol (2010) 3:291–300. 10.1038/mi.2010.6 20404812 PMC2875872

[267] Paul WE Seder RA Lymphocyte responses and cytokines. Cell (1994) 76:241–51. 10.1016/0092-8674(94)90332-8 7904900

[268] Bonizzi G Karin M The two NF-kappaB activation pathways and their role in innate and adaptive immunity. Trends Immunol (2004) 25:280–8. 10.1016/j.it.2004.03.008 15145317

[269] Schindler C Levy DE Decker T JAK-STAT signaling: from interferons to cytokines. J Biol Chem (2007) 282:20059–63. 10.1074/jbc.R700016200 17502367

[270] Borden EC Williams BR. Interferon-stimulated genes and their protein products: what and how? J Interferon Cytokine Res (2011) 31:1–4. 10.1089/jir.2010.0129 21226605

[271] Furuya Y Steiner D Metzger DW Does type I interferon limit protective neutrophil responses during pulmonary *Francisella tularensis* infection? Front Immunol (2014) 5:355 10.3389/fimmu.2014.00355 25101094 PMC4107939

[272] Garcia-Sastre A. Induction and evasion of type I interferon responses by influenza viruses. Virus Res (2011) 162:12–8. 10.1016/j.virusres.2011.10.017 22027189 PMC3640439

[273] Martinelli S Urosevic M Daryadel A Oberholzer PA Baumann C Fey MF Induction of genes mediating interferon-dependent extracellular trap formation during neutrophil differentiation. J Biol Chem (2004) 279:44123–32. 10.1074/jbc.M405883200 15302890

[274] Meunier I von Messling V. NS1-mediated delay of type I interferon induction contributes to influenza A virulence in ferrets. J Gen Virol (2011) 92:1635–44. 10.1099/vir.0.032193-0 21411677

[275] Seo SU Kwon HJ Ko HJ Byun YH Seong BL Uematsu S Type I interferon signaling regulates Ly6C(hi) monocytes and neutrophils during acute viral pneumonia in mice. PLoS Pathog (2011) 7:e1001304. 10.1371/journal.ppat.1001304 21383977 PMC3044702

[276] Svitek N Rudd PA Obojes K Pillet S von Messling V. Severe seasonal influenza in ferrets correlates with reduced interferon and increased IL-6 induction. Virology (2008) 376:53–9. 10.1016/j.virol.2008.02.035 18420248

[277] Wang X Li M Zheng H Muster T Palese P Beg AA Influenza A virus NS1 protein prevents activation of NF-kappaB and induction of alpha/beta interferon. J Virol (2000) 74:11566–73. 10.1128/JVI.74.24.11566-11573.2000 11090154 PMC112437

[278] Garcia-Romo GS Caielli S Vega B Connolly J Allantaz F Xu Z Netting neutrophils are major inducers of type I IFN production in pediatric systemic lupus erythematosus. Sci Transl Med (2011) 3:73ra20. 10.1126/scitranslmed.3001201 21389264 PMC3143837

[279] Decker P Neutrophils and interferon-alpha-producing cells: who produces interferon in lupus? Arthritis Res Ther (2011) 13:118 10.1186/ar3345 21745418 PMC3239333

[280] Stock AT Smith JM Carbone FR. Type I IFN suppresses Cxcr2 driven neutrophil recruitment into the sensory ganglia during viral infection. J Exp Med (2014) 211:751–9. 10.1084/jem.20132183 24752295 PMC4010892

[281] Xin L Vargas-Inchaustegui DA Raimer SS Kelly BC Hu J Zhu L Type I IFN receptor regulates neutrophil functions and innate immunity to *Leishmania* parasites. J Immunol (2010) 184:7047–56. 10.4049/jimmunol.0903273 20483775 PMC4159077

[282] Durbin JE Fernandez-Sesma A Lee CK Rao TD Frey AB Moran TM Type I IFN modulates innate and specific antiviral immunity. J Immunol (2000) 164:4220–8. 10.4049/jimmunol.164.8.4220 10754318

[283] Hashimoto Y Moki T Takizawa T Shiratsuchi A Nakanishi Y. Evidence for phagocytosis of influenza virus-infected, apoptotic cells by neutrophils and macrophages in mice. J Immunol (2007) 178:2448–57. 10.4049/jimmunol.178.4.2448 17277152

[284] Tate MD Brooks AG Reading PC Mintern JD. Neutrophils sustain effective CD8(+) T-cell responses in the respiratory tract following influenza infection. Immunol Cell Biol (2012) 90:197–205. 10.1038/icb.2011.26 21483446

[285] Tate MD Deng YM Jones JE Anderson GP Brooks AG Reading PC. Neutrophils ameliorate lung injury and the development of severe disease during influenza infection. J Immunol (2009) 183:7441–50. 10.4049/jimmunol.0902497 19917678

[286] Herold S Tabar TS Janssen H Hoegner K Cabanski M Lewe-Schlosser P Exudate macrophages attenuate lung injury by the release of IL-1 receptor antagonist in gram-negative pneumonia. Am J Respir Crit Care Med (2011) 183:1380–90. 10.1164/rccm.201009-1431OC 21278303

[287] Dean RA Cox JH Bellac CL Doucet A Starr AE Overall CM. Macrophage-specific metalloelastase (MMP-12) truncates and inactivates ELR+ CXC chemokines and generates CCL2, -7, -8, and -13 antagonists: potential role of the macrophage in terminating polymorphonuclear leukocyte influx. Blood (2008) 112:3455–64. 10.1182/blood-2007-12-129080 18660381

[288] Fadok VA Bratton DL Konowal A Freed PW Westcott JY Henson PM. Macrophages that have ingested apoptotic cells in vitro inhibit proinflammatory cytokine production through autocrine/paracrine mechanisms involving TGF-beta, PGE2, and PAF. J Clin Invest (1998) 101:890–8. 10.1172/JCI1112 9466984 PMC508637

[289] El Kebir D Filep JG. Targeting neutrophil apoptosis for enhancing the resolution of inflammation. Cells (2013) 2:330–48. 10.3390/cells2020330 24709704 PMC3972676

[290] Godson C Mitchell S Harvey K Petasis NA Hogg N Brady HR. Cutting edge: lipoxins rapidly stimulate nonphlogistic phagocytosis of apoptotic neutrophils by monocyte-derived macrophages. J Immunol (2000) 164:1663–7. 10.4049/jimmunol.164.4.1663 10657608

[291] Poe SL Arora M Oriss TB Yarlagadda M Isse K Khare A STAT1-regulated lung MDSC-like cells produce IL-10 and efferocytose apoptotic neutrophils with relevance in resolution of bacterial pneumonia. Mucosal Immunol (2013) 6:189–99. 10.1038/mi.2012.62 22785228 PMC3505806

[292] Dienz O Rud JG Eaton SM Lanthier PA Burg E Drew A Essential role of IL-6 in protection against H1N1 influenza virus by promoting neutrophil survival in the lung. Mucosal Immunol (2012) 5:258–66. 10.1038/mi.2012.2 22294047 PMC3328598

[293] Arndt U Wennemuth G Barth P Nain M Al-Abed Y Meinhardt A Release of macrophage migration inhibitory factor and CXCL8/interleukin-8 from lung epithelial cells rendered necrotic by influenza A virus infection. J Virol (2002) 76:9298–306. 10.1128/JVI.76.18.9298-9306.2002 12186913 PMC136427

[294] Simard JC Girard D Tessier PA. Induction of neutrophil degranulation by S100A9 via a MAPK-dependent mechanism. J Leukoc Biol (2010) 87:905–14. 10.1189/jlb.1009676 20103766

[295] Tsai SY Segovia JA Chang TH Morris IR Berton MT Tessier PA DAMP molecule S100A9 acts as a molecular pattern to enhance inflammation during influenza A virus infection: role of DDX21-TRIF-TLR4-MyD88 pathway. PLoS Pathog (2014) 10:e1003848. 10.1371/journal.ppat.1003848 24391503 PMC3879357

[296] Camp JV Critical Insights into the Pathogenesis of Clinical Isolates of Pandemic Influenza A(H1N1) 2009 Virus in Mouse and Ferret Models [Doctoral Electronic Theses and Dissertations]. Louisville, KY: University of Louisville (2015).

