# Supplementary material for: A Role for Neutrophils in Viral Respiratory Disease
Source: Front Immunol. 2017 May 12;8:550. doi: 10.3389/fimmu.2017.00550 (PMC5427094; doi:10.3389/fimmu.2017.00550)
Supplement: Supplementary file 1 [file Table_1.DOCX]

Supplementary Material

A Role for Neutrophils in Viral Respiratory Disease

Jeremy V. Camp, Colleen B. Jonsson*

*** Correspondence:** Colleen Jonssson: cjonsson@utk.edu

# Supplementary Data

Animal models have played an essential role in studying viral respiratory disease pathogenesis. Hypotheses about the relative contribution of specific host factors to disease progression can be tested via antibody-mediated cell depletion and/or through the use of gene recombinant technology to create a “knock-out” laboratory mouse. Below are tables summarizing studies using these techniques to investigate the innate immune response to influenza A virus in mice. Specific attention is given to the effect of the experimental manipulation on the neutrophil response relative to infected wild-type mice. Mice are not natural hosts of influenza A viruses, and therefore the viruses must be adapted to produce viral infection models of the desired phenotype – the most common of which is the “PR8” mouse-adapted H1N1 A/Puerto Rico/8/1934 virus. Thus, the tables below include whether the model is a “lethal” model of infection (and therefore determine if the mice are rescued by the experimental manipulation) as well as the viral dose, although this is less informative due to the inconsistent concentration determination across studies (i.e., MLD_50_, TCID_50_, pfu, etc. as described in the tables below).

Table 1. Effect of cytokine receptor knockout mice infected with IAV on neutrophils

| **Mouse Model** | **Virus** | **Lethal model** | **Dose** | **Effect on Lung Neutrophils** | **Effect on Disease Phenotype compared to wt** | **Ref** |
| --- | --- | --- | --- | --- | --- | --- |
| IFNαβR^-/-^ | H1N1  A/PR/8/1934 | Yes | 6.7 LD_50_ | Same as wt | Same as wt | (1) |
| IFNαβR^-/-^ | H3N2 X31 (A/PR/8/1934) | Yes | 800 TCID_50_ | Reduced | Protection | (2) |
| IFNαβR^-/-^ | H1N1  A/California/04/2009 | Yes | 330 TCID_50_ | Reduced | Protection | (2) |
| IFNαβR^-/-^ | H1N1  A/PR/8/1934 | LD_50_ | 10000 PFU | Increased | Increased lethality | (3) |
| IL-1R1^-/-^ | H5N1  A/HK/483/1997 | Yes | 10^3^ MID_50_ | Increased | Delayed Death | (4) |
| IL-1R1^-/-^ | H5N1  A/HK/483/1997 | Yes | 10^3^ MID_50_ | Increased | Delayed Death | (4) |
| IL-1R1^-/-^ | H5N1  A/HK/483/1997 | Yes | 10^3^ MID_50_ | Same as wt | Same as wt | (5) |
| IL-1R1^-/-^ | H5N1  A/HK/486/1997 | No | 10^3^ MID_50_ | Slightly less (n.s.) | Reduced Weight loss | (5) |
| IL1-R1^-/-^ | H1N1  A/PR/8/1934 | No | 100 PFU | Reduced | Increased Lethality | (6) |
| IL1-R1^-/-^ | H1N1  A/PR/8/1934 | No | 100 PFU | Reduced | Increased Lethality | (6) |
| IL1-R1^-/-^ | H1N1 r1918 | Yes | 10^6^ PFU | Same as wt | Increased Complement Gene Expression | (7) |
| IL-6R^-/-^ | H1N1  A/PR/8/1934 | No | 3000 EUI | n/c | Increased Lethality | (8) |
| IL-17RA^-/-^ | H1N1  A/PR/8/1934 | Yes | 1 LD_90_ | Reduced | Protection | (9) |
| TNF-R1^-/-^ | H5N1  A/Hong Kong/483/1997 | Yes | 10^3^ MID_50_ | Reduced | Delayed Death | (4) |
| TNF-R1^-/-^ | H5N1  A/Hong Kong/483/1997 | Yes | 10^3^ MID_50_ | n/c | Reduced leukopenia | (5) |
| TNF-R1^-/-^ | H5N1  A/Vietnam/1203/04 | Yes | 10^3^ EID_50_ | n/c | Same as wt | (10) |
| TNF-R1^-/-^ TNF-R2^-/-^ | H5N1  A/Vietnam/1203/04 | Yes | 10^3^ EID_50_ | n/c | Increased survival (n.s.) | (10) |
| TNF-R1^-/-^ TNF-R2^-/-^ | H1N1 r1918 | Yes | 10^6^ PFU | Reduced | Delayed death | (7) |
| TNF-R1^-/-^ TNF-R2^-/-^ IL1R^-/-^ | H5N1  A/Hong Kong/483/1997 | Yes | 10^3^ MID_50_ | Reduced | Delayed Death | (4) |

wt refers to wild-type mice; LD_50_ = 50% mouse lethal dose; TCID_50_ = 50% tissue culture infectious dose; PFU = plaque forming units; EIU = egg infectious units; MID_50_ = 50% mouse infectious dose; EID_50_ = 50% egg infectious dose; n/c = no change. PR = Puerto Rico, HK = Hong Kong, r1918 = strain of H1N1 Influenza A virus from 1918 rescued via reverse genetics techniques.

Table 2. Effect of chemokine receptor knockout mice infected with IAV on neutrophils

| **Mouse Model** | **Virus** | **Lethal model** | **Dose** | **Effect on Lung Neutrophils** | **Effect on Disease Phenotype compared to wt** | **Ref** |
| --- | --- | --- | --- | --- | --- | --- |
| CCR2^-/-^ | H1N1 A/PR/8/1934 | No | 3.3 PFU | Increased | Increased lung CCL2, CCL3, CCL4, CXCL1, CXCL2, CXCL10 | (11) |
| CCR2^-/-^ | H1N1 A/PR/8/1934 | 70-80% Survival | 5 HAU | Increased, delayed | Reduced lethality, increased virus in lung, increased CCL2, CXCL10 in lung | (12) |
| CCR5^-/-^ | H1N1 A/PR/8/1934 | 70-80% Survival | 5 HAU | Increased | Increased lethality, increased virus in lung, , increased CCL2, CCL5, CXCL10 in lung | (12) |
| CCR2^-/-^  CCL3^-/-^ | H1N1 A/PR/8/1934 | 70-80% Survival | 5 HAU | n/c | Reduced lethality, increased virus in lungs | (12) |
| CXCR2^-/-^ | H1N1 A/PR/8/1934 | No | 10-100 PFU | Reduced | Increased lung macrophages  Decreased T cell recruitment | (13) |
| CXCR3^-/-^ | H1N1 A/PR/8/1934 | No | 3.3 PFU | Reduced | Same as wt | (14) |
| CXCR3^-/-^ | H1N1 A/PR/8/1934 | Yes | 6.7 LD_50_ | Reduced | Increased Survival | (1) |

wt refers to wild-type mice; PFU = plaque forming units; HAU = hemagglutinating units, LD_50_ = 50% mouse lethal dose; n/c = no change, PR = Puerto Rico

Table 3. Effect of cytokine knockout mice infected with IAV on neutrophils

| **Mouse Model** | **Virus** | **Lethal model** | **Dose** | **Effect on Lung Neutrophils** | **Effect on Disease Phenotype compared to wt** | **Ref** |
| --- | --- | --- | --- | --- | --- | --- |
| IL-1β^-/-^ | H3N2 A/Taiwan/3446/2002 | No | 5x10^5^ PFU | Same as wt | Same as wt | (15) |
| IL-1β^-/-^ | H3N2 A/TW/3446/02  with  PA gene from A/VN/1194/04 | No | 5x10^5^ PFU | Same as wt | Delayed pathology | (15) |
| IL-1β (Casp 1^-/-^) | H3N2 A/Taiwan/3446/2002 | No | 5x10^5^ PFU | Same as wt | Same as wt | (15) |
| IL-1β (Casp 1^-/-^) | H3N2 A/TW/3446/02  with  PA gene from A/VN/1194/04 | No | 5x10^5^ PFU | Increased | Weight loss, delayed pathology | (15) |
| IL-1β (Casp 1^-/-^) | H1N1 A/PR/8/1934 | 70% Survival | 6x10^4^ PFU with 0.1% alum | Reduced | Increased Lethality | (16) |
| IL-1β (Casp 1^-/-^) | H1N1 A/PR/8/1934 | 70-80% Survival | 8000 EID_50_ | Reduced | Increased Lethality | (17) |
| IL-6^-/-^ | H5N1 A/Hong Kong/483/1997 | Yes | 10^3^ MID_50_ | n/c | Same as wt | (5) |
| IL-6^-/-^ | H5N1 A/Hong Kong/486/1997 | No | 10^3^ MID_50_ | n/c | Same as wt | (5) |
| IL-6^-/-^ | H5N1 A/Vietnam/1203/04 | Yes | 10^3^ EID_50_ | n/c | Same as wt | (10) |
| IL-6^-/-^ | H1N1 A/PR/8/1934 | No | 3000 EIU | Decreased | Features of chronic infection,  delayed viral clearance | (8) |
| TNF^-/-^ | H5N1 A/Vietnam/1203/04 | Yes | 10^3^ EID_50_ | n/c | Same as wt | (10) |
| IL-18^-/-^ | H3N2 X31 (A/PR/8/1934) | No | 10^4^ PFU | Increased | Increased virus in lungs early, cleared virus same as wt | (18) |

wt = wild-type mice; Casp 1 = caspase 1; PFU = plaque forming units; EID_50_ = 50% egg infectious dose; MID_50_ = 50% mouse infectious dose; EIU = egg infectious units, n/c = no change.

Table 4. Effect of chemokine knockout mice infected with IAV on neutrophils

| **Mouse Model** | **Virus** | **Lethal model** | **Dose** | **Effect on Lung Neutrophils** | **Effect on Disease Phenotype compared to wt** | **Ref** |
| --- | --- | --- | --- | --- | --- | --- |
| CCL2^-/-^ | H5N1 A/Vietnam/1203/04 | Yes | 10^3^ EID_50_ | n/c | Same as wt | (10) |
| CCL3^-/-^ | H5N1 A/Hong Kong/483/1997 | Yes | 10^3^ MID_50_ | n/c | Same as wt | (5) |
| CCL3^-/-^ | H5N1 A/Hong Kong/486/1997 | No | 10^3^ MID_50_ | n/c | Same as wt | (5) |
| CCL5^-/-^ | H1N1 A/PR/8/1934 | No | 3.3 PFU | Same as wt | Same as wt | (14) |
| CXCL10^-/-^ | H1N1 A/PR/8/1934 | Yes | 6.7 LD_50_ | Reduced | Increased Survival | (1) |

wt = wild-type mice; PFU = plaque forming units; EID_50_ = 50% egg infectious dose; MID_50_ = 50% mouse infectious dose; LD_50_ = 50% mouse lethal dose, n/c = no change. PR = Puerto Rico.

Table 5. Effect of neutrophil effector knockout mice on IAV infection

| **Mouse Model** | **Virus** | **Lethal model** | **Dose** | **Effect on Lung Neutrophils** | **Effect on Disease Phenotype compared to wt** | **Ref** |
| --- | --- | --- | --- | --- | --- | --- |
| Cybb^tm1^ Mice (gp91^phox^ deficient) | H3N2 X31 (A/PR/8/1934) | No | 50 HAU | Increased | Increased lung cellularity, Decreased lung consolidation,  Increased TNFa, IL6, CCL2,  Decreased virus in lung | (19) |
| Elane^-/-^ | H3N2* A/Memphis/1/1971  Written as H3N1 in pub. | No | 10^4.5^ PFU | Same as wt | Same as wt | (20) |
| NETs  (PAD4^-/-^) | H1N1 A/WSN/33 | 50% Survive | 1 LD_50_ | Same as wt | Same as wt | (21) |

wt = wild-type mice; HAU = hemagglutinating units; PFU = plaque forming units; LD_50_= mouse 50% lethal dose, n/c = no change.

# REFERENCES

1. **Ichikawa A, Kuba K, Morita M, Chida S, Tezuka H, Hara H, Sasaki T, Ohteki T, Ranieri VM, dos Santos CC, Kawaoka Y, Akira S, Luster AD, Lu B, Penninger JM, Uhlig S, Slutsky AS, Imai Y.** 2013. CXCL10-CXCR3 enhances the development of neutrophil-mediated fulminant lung injury of viral and nonviral origin. Am J Respir Crit Care Med **187:**65-77.

2. **Davidson S, Crotta S, McCabe TM, Wack A.** 2014. Pathogenic potential of interferon alphabeta in acute influenza infection. Nat Commun **5:**3864.

3. **Seo SU, Kwon HJ, Ko HJ, Byun YH, Seong BL, Uematsu S, Akira S, Kweon MN.** 2011. Type I interferon signaling regulates Ly6C(hi) monocytes and neutrophils during acute viral pneumonia in mice. PLoS Pathog **7:**e1001304.

4. **Perrone LA, Szretter KJ, Katz JM, Mizgerd JP, Tumpey TM.** 2010. Mice lacking both TNF and IL-1 receptors exhibit reduced lung inflammation and delay in onset of death following infection with a highly virulent H5N1 virus. J Infect Dis **202:**1161-1170.

5. **Szretter KJ, Gangappa S, Lu X, Smith C, Shieh WJ, Zaki SR, Sambhara S, Tumpey TM, Katz JM.** 2007. Role of host cytokine responses in the pathogenesis of avian H5N1 influenza viruses in mice. J Virol **81:**2736-2744.

6. **Schmitz N, Kurrer M, Bachmann MF, Kopf M.** 2005. Interleukin-1 is responsible for acute lung immunopathology but increases survival of respiratory influenza virus infection. J Virol **79:**6441-6448.

7. **Belisle SE, Tisoncik JR, Korth MJ, Carter VS, Proll SC, Swayne DE, Pantin-Jackwood M, Tumpey TM, Katze MG.** 2010. Genomic profiling of tumor necrosis factor alpha (TNF-alpha) receptor and interleukin-1 receptor knockout mice reveals a link between TNF-alpha signaling and increased severity of 1918 pandemic influenza virus infection. J Virol **84:**12576-12588.

8. **Dienz O, Rud JG, Eaton SM, Lanthier PA, Burg E, Drew A, Bunn J, Suratt BT, Haynes L, Rincon M.** 2012. Essential role of IL-6 in protection against H1N1 influenza virus by promoting neutrophil survival in the lung. Mucosal Immunol **5:**258-266.

9. **Crowe CR, Chen K, Pociask DA, Alcorn JF, Krivich C, Enelow RI, Ross TM, Witztum JL, Kolls JK.** 2009. Critical role of IL-17RA in immunopathology of influenza infection. J Immunol **183:**5301-5310.

10. **Salomon R, Hoffmann E, Webster RG.** 2007. Inhibition of the cytokine response does not protect against lethal H5N1 influenza infection. Proc Natl Acad Sci U S A **104:**12479-12481.

11. **Wareing MD, Lyon A, Inglis C, Giannoni F, Charo I, Sarawar SR.** 2007. Chemokine regulation of the inflammatory response to a low-dose influenza infection in CCR2-/- mice. J Leukoc Biol **81:**793-801.

12. **Dawson TC, Beck MA, Kuziel WA, Henderson F, Maeda N.** 2000. Contrasting effects of CCR5 and CCR2 deficiency in the pulmonary inflammatory response to influenza A virus. Am J Pathol **156:**1951-1959.

13. **Wareing MD, Shea AL, Inglis CA, Dias PB, Sarawar SR.** 2007. CXCR2 is required for neutrophil recruitment to the lung during influenza virus infection, but is not essential for viral clearance. Viral Immunol **20:**369-378.

14. **Wareing MD, Lyon AB, Lu B, Gerard C, Sarawar SR.** 2004. Chemokine expression during the development and resolution of a pulmonary leukocyte response to influenza A virus infection in mice. J Leukoc Biol **76:**886-895.

15. **Huang CH, Chen CJ, Yen CT, Yu CP, Huang PN, Kuo RL, Lin SJ, Chang CK, Shih SR.** 2013. Caspase-1 deficient mice are more susceptible to influenza A virus infection with PA variation. J Infect Dis **208:**1898-1905.

16. **Allen IC, Scull MA, Moore CB, Holl EK, McElvania-TeKippe E, Taxman DJ, Guthrie EH, Pickles RJ, Ting JP.** 2009. The NLRP3 inflammasome mediates in vivo innate immunity to influenza A virus through recognition of viral RNA. Immunity **30:**556-565.

17. **Thomas PG, Dash P, Aldridge JR, Jr., Ellebedy AH, Reynolds C, Funk AJ, Martin WJ, Lamkanfi M, Webby RJ, Boyd KL, Doherty PC, Kanneganti TD.** 2009. The intracellular sensor NLRP3 mediates key innate and healing responses to influenza A virus via the regulation of caspase-1. Immunity **30:**566-575.

18. **Liu B, Mori I, Hossain MJ, Dong L, Takeda K, Kimura Y.** 2004. Interleukin-18 improves the early defence system against influenza virus infection by augmenting natural killer cell-mediated cytotoxicity. J Gen Virol **85:**423-428.

19. **Snelgrove RJ, Edwards L, Rae AJ, Hussell T.** 2006. An absence of reactive oxygen species improves the resolution of lung influenza infection. Eur J Immunol **36:**1364-1373.

20. **Foong RE, Sly PD, Larcombe AN, Zosky GR.** 2010. No role for neutrophil elastase in influenza-induced cellular recruitment, cytokine production or airway hyperresponsiveness in mice. Respir Physiol Neurobiol **173:**164-170.

21. **Hemmers S, Teijaro JR, Arandjelovic S, Mowen KA.** 2011. PAD4-mediated neutrophil extracellular trap formation is not required for immunity against influenza infection. PLoS One **6:**e22043.
